# Transgenic Mice Expressing Human α-Synuclein 1-103 Fragment as a Novel Model of Parkinson’s Disease

**DOI:** 10.3389/fnagi.2021.760781

**Published:** 2021-10-22

**Authors:** Ye Tian, Mingyang He, Lina Pan, Xin Yuan, Min Xiong, Lanxia Meng, Zhaohui Yao, Zhui Yu, Keqiang Ye, Zhentao Zhang

**Affiliations:** ^1^Department of Neurology, Renmin Hospital of Wuhan University, Wuhan, China; ^2^Department of Geriatrics, Renmin Hospital of Wuhan University, Wuhan, China; ^3^Department of Critical Care Medicine, Renmin Hospital of Wuhan University, Wuhan, China; ^4^Department of Pathology and Laboratory Medicine, Emory University School of Medicine, Atlanta, GA, United States

**Keywords:** Parkinson’s disease, α-synuclein, asparagine endopeptidase, mouse model, neurodegenerative disease

## Abstract

Parkinson’s disease (PD) is one of the most common neurodegenerative disorders. However, its cellular and molecular mechanisms still wrap in the mist. This is partially caused by the absence of appropriate animal models mimicking sporadic PD that constitutes the majority of cases. Previously, we reported that a cysteine protease, asparagine endopeptidase (AEP), is activated in an age-dependent manner, and cleaves α-synuclein in the brain of sporadic PD patients. The AEP-derived α-synuclein 1-103 fragment is required for the pathogenesis of PD. Thus, we designed and characterized a novel transgenic mouse line expressing α-synuclein 1-103 (designated N103 mice). This model shows an abundant accumulation of pathological α-synuclein in the central nervous system, loss of dopaminergic neurons in the substantia nigra, and progressive striatal synaptic degeneration. The N103 mice also manifest age-dependent PD-like behavioral impairments. Notably, the mice show weight loss and constipation, which are the common non-motor symptoms in PD. The RNA-sequencing analysis found that the transcriptomics pattern was extensively altered in N103 mice. In conclusion, the N103 mouse line, as a brand-new tool, might provide new insights into PD research.

## Introduction

Parkinson’s disease (PD) is the second most frequent neurodegenerative disorder, affecting 6.1 million individuals globally (Armstrong and Okun, 2020). It is pathologically characterized by the selective vulnerability of dopaminergic neurons in the substantia nigra pars compacta and progressive accumulation of Lewy bodies (LBs) or Lewy neurites (LNs) mainly consisting of misfolded α-synuclein (Goedert, 2015). The available therapies can only partially alleviate the motor symptoms but fail to stop or reverse the progression of neurodegeneration (Okun, 2012, 2017; Bressman and Saunders-Pullman, 2019). The underlying mechanisms of PD remain poorly understood. α-Synuclein is believed to be the key player in the onset and development of PD. Multiplications of the α-synuclein coding gene (SNCA) and several point mutations (e.g., A53T, A30P, and E46K) have been identified to cause familial PD (Blesa, 2018). However, familial PD makes up only 5%-10% of the total cases. The sporadic form constitutes the majority of PD cases (Balestrino and Schapira, 2020).

α-Synuclein is one of the most abundant proteins in the brain. The molecular mechanisms that mediate its aggregation and toxicity in sporadic PD remain to be elucidated. Previously, we reported a novel cysteine protease, asparagine endopeptidase (AEP) that contributes to the aggregation of α-synuclein in PD. AEP is activated in the brains of AD patients during aging. The aberrantly activated AEP cleaves α-synuclein at N103 and generates the α-synuclein 1-103 fragments. This N-terminal fragment of α-synuclein is prone to aggregate, shows significant neurotoxicity, and induces PD-like pathology when expressed in mouse brains. Blocked of AEP-mediated generation of the α-synuclein 1-103 fragment attenuated the aggregation of α-synuclein and PD-like behavioral impairments in a mouse model of PD (Zhang et al., 2017a). Besides, α-synuclein 1-103 forms mixed fibers by interacting with tau, inducing dopaminergic neuronal death in the substantia nigra (Ahn et al., 2020). These results indicate that α-synuclein 1-103 fragment is involved in the pathogenesis of PD.

Many animal models have been developed for investigating the pathogenesis of PD or assessing the effectiveness of tentative therapies. Toxin-induced mouse models are wildly used. 1-methyl-4-phenyl-1,2,3,6-tetrahydropyridine (MPTP), 6-hydroxydopamine (6-OHDA), rotenone, and paraquat are administered systemically or stereoscopically to selectively impair the dopaminergic nigrostriatal pathway (Perier et al., 2003; Jackson-Lewis and Przedborski, 2007; Tieu, 2011; Lu et al., 2014; Jagmag et al., 2016). Various transgenic mouse lines also have been constructed. Transgenic mouse models that carry PD-associated genes have been extensively used for decades. The most popular one is the α-synuclein A53T transgenic mice which carry the A53T mutated human α-synuclein (Line M83). After the prion hypothesis of neurodegenerative disorders was proposed, non-transgenic PD models generated by inoculating preformed fibrils of α-synuclein or brain lysate from patients with synucleinopathy were developed as well (Luk et al., 2012; Masuda-Suzukake et al., 2013; Recasens et al., 2014; Peelaerts et al., 2015).

Although many PD mouse models have been established, a proper model that mimics sporadic PD is to be developed yet. As for these current PD models, either they are induced by the inhalation of neurotoxins or α-synuclein fibrils, which cannot reflect the authentic pathological origin in the real world, or they are generated by deliberately importing identified mutations, which mimic what happens in familial PD. Since we found that AEP-mediated production of the α-synuclein 1-103 fragment is involved in the onset and progression of sporadic PD, we developed a transgenic mouse line expressing α-synuclein 1-103 (designated N103 mice) to assess its capability as a candidate model for PD. This model manifests stable and progressive neurodegeneration that covers abundant PD-like pathological features such as the accumulation of α-synuclein in the central nervous system, degeneration of dopaminergic neurons in the substantia nigra, and a significant loss of striatal synapses at the age of 9 months. Motor symptoms also emerge in an age-dependent manner. Furthermore, this model well-replicates some non-motor symptoms of PD including weight loss and constipation at an early stage. Thus, this newly designed N103 mouse line is qualified to be a novel tool for sporadic PD-related research. It will serve as a novel tool for drug screening and lay a foundation for elucidating the underlying molecular and cellular mechanisms of PD.

## Materials and Methods

### Generation, Identification, and Handing of N103 Mice

The human α-synuclein 1-103 gene prefixed with Thy1 promoter was imported into C57BL/6J mouse line using fertilized egg injection following the protocol described before (Giasson et al., 2002). According to this approach, the transgene would be randomly inserted into the mouse genome. Linear fragments harboring Thy1 promoter and the human α-synuclein 1-103 cDNAs together with its 5’ untranslated region (UTR) containing a Kozak enhance sequence before the initial codon and the 3’ PolyA tail were cloned and microinjected into C57BL/6J mouse eggs under the help of Biomedical Research Institute of Nanjing University. After embryo transplantation, mice were obtained and authenticated by quantitative polymerase chain reaction (qPCR). Two of them identified as high-expressing individuals were consecutively crossbred with WT mice to acquire a stably inherited heterozygote strain and the homozygotes were obtained by crossbreeding heterozygotes. Genotyping on offspring was routinely implemented with tail DNA samples utilizing PCR and qPCR. The PCR primers were: Target sequence forward: 5′-GTAATGAAGTCACCCAGCAGGG-3′, reverse: 5′-TACTGCTGTCACACCCGTCACC-3′; β-actin forward: 5′-CT AGGCCACAGAATTGAAAGATCT-3′, reverse: 5′-GTAGGTG GAAATTCTAGCATCATCC-3′. The qPCR primers were: Target sequence forward: 5′-CAAGGAGGGAGTTGTGGCTG-3′, reverse: 5′-GGAGCCTACATAGAGAACACCCTC-3′; β-actin forward: 5′-TCACCAGTCATTTCTGCCTTTG-3′, reverse: 5′-C ACGTGGGCTCCAGCATT-3′. Mice identified negative were used as littermate control. All animals were handed in strict accordance with the Experimental Animal Management Criterion of the Renmin Hospital of Wuhan University.

### Tissue Preparation

Mice were anesthetized using 2% pentobarbital sodium and sequentially perfused with adequate saline and 4% paraformaldehyde for immunohistochemistry and thioflavin-S staining. Tissues of interest were removed, infiltrated with paraffin, and cut into serial sections. For biochemical analysis, mice were perfused with ice-cold saline only. Brains were rapidly separated and stored at −80°C.

### Western Blot Analysis

Mouse brains were extracted in RIPA buffer (Cat# P0013B, Beyotime) supplemented with protease inhibitor cocktail and phosphatase inhibitor cocktail B (Cat# P1086, Beyotime) and followed by centrifugation (12,000 r.p.m., 4°C, 20 min). Total protein contents of supernatants were quantified by BCA assay before being boiled in loading buffer. After SDS-PAGE, proteins were transferred onto a nitrocellulose membrane and incubated at 4°C overnight with appropriate primary antibodies: anti-α-synuclein 1-103 rabbit polyclonal antibody (1:1,000, Ye’s lab), anti-phospho-α-synuclein (Ser129) (D1R1R) antibody (1:1,000, Cat# 23706, Cell Signaling Technology), anti-tyrosine hydroxylase antibody (1:1,000, Cat# AB152, Millipore), purified mouse anti-α-synuclein Syn-1/Clone 42 antibody (1:1000, Cat# 610786, BD Transduction Laboratories), and HRP-conjugated GAPDH monoclonal antibody (1:10,000, Cat# HRP-60004, Proteintech). The effectiveness of anti-α-synuclein 1-103 rabbit polyclonal antibody has been identified in our previous report (Zhang et al., 2017a).

### Immunohistochemistry

Sections were deparaffinized in xylene, hydrated through descending ethanol. After being steeped in antigen retrieval solution (80 mM citric acid, 20 mM sodium citrate, pH 6.0) at 94°C for 20 min, sections were incubated in 3% hydrogen peroxide for 10 min to eliminate endogenous peroxidase activity. After blocked in 3% BSA for 30 min, sections were incubated at 4°C overnight with primary antibodies: anti-α-synuclein 1-103 rabbit polyclonal antibody (1:1,000), anti-α-synuclein phospho (pSer129) antibody (1:500, Cat# MMS-5091, BioLegend), anti-tyrosine hydroxylase antibody (1:1,000, Cat# AB152, Millipore), anti-MAP2 antibody (1:500, Cat# 17490-1-AP, Proteintech), anti-Iba1 antibody (1:1,000, Cat# 17198, Cell Signaling Technology), anti-GFAP antibody (1:500, Cat# 12389, Cell Signaling Technology), anti-Olig2 antibody (1:500, Cat# 13999-1-AP, Proteintech), anti-ubiquitin antibody (1:200, Cat# A19686, ABclonal), and recombinant anti-α-synuclein aggregate antibody [MJFR-14-6-4-2] - conformation-specific (1:5000, Cat# ab209538, Abcam). Signals were developed utilizing the DAB staining kit (Absin, Cat# abs957). For double immunofluorescence staining, a mixture of Alexa Fluor 488- and 568-coupled secondary antibodies were applied. Cell nuclei were labeled with DAPI. Images were captured using an Olympus DP80 microscope equipped with TH4-200 and U-HGLGPS light sources. Quantification of the integrated fluorescence intensity was analyzed after binarization with a fixed proper brightness threshold (48 to 255) for baseline correction utilizing the ImageJ software (version 2.1.0/1.53c).

### Thioflavin-S Staining

Sections were incubated in filtered thioflavin-S solution (0.05% w/v in 50% ethanol) for 10 min at room temperature, then treated with 80% ethanol for 15 s and washed in PBS three times.

### Stereological Cell Counting

We used the Olympus DP80 microscope and its matched software (cellSens Ver1.7.1/1.8/1.9 controlling DP80) for cell counting. Each group contains 5 mice, and the entire substantia nigra of each mouse was continuously sliced into 4 μm-thick paraffin sections (100∼150 sections for each mouse). Every 6th section was involved in the counting procedure (about 20 sections for each mouse). Since every 6th section determined a volume with a thickness of 24 μm, which is close to the mean diameter of the cell body of dopaminergic neurons, the sum of all involved sections’ cell numbers was considered as the final result of a certain mouse.

### Optical Densitometry Analysis

Striatal TH-positive fiber density was measured following a procedure in the previous literature (Decressac et al., 2011). The integrated optical density of the striatum at six coronal levels of equal distance relative to bregma was measured using the ImageJ software (version 2.1.0/1.53c) and the IHC Profiler plug-in according to its official guideline. The staining signal was calibrated by subtracting the baseline of the cortex.

### High-Performance Liquid Chromatography Analysis

The striatum was separated and snap-frozen in liquid nitrogen. Before analysis, tissues were weighed and homogenized in 0.1 M perchloric acid solution containing 0.1% cysteine. After centrifugation (14,000 r.p.m., 4°C, 15 min), supernatants were filtered and analyzed by HPLC. Prepared samples were injected onto a chromatographic column (4.6 mm × 150 mm) and separated by a mobile phase (8% methanol in 3 mM sodium heptanesulfonate, 100 mM sodium acetate, 85 mM citric acid, 0.2 mM EDTA) at a flow rate of 1 ml/min. DA, DOPAC, and HVA contents within each sample were quantified by comparison to the standard specimens.

### Transmission Electron Microscopy

Striatal synaptic density was determined by electron microscopy as previously described (Zhang et al., 2017b). After deep anesthesia, animals were perfused with 2% glutaraldehyde. The striatum was dissected and post-fixed at 4°C shielded from light until further preparation. After the standard procedure was performed, ultrathin sections were treated with 2% uranyl acetate, lead acetate, and viewed at 100 kV under a transmission electron microscope (HT7800/HT7700, Hitachi). The synaptic function was assessed by the density of synaptic vesicles and synapses.

### RNA Sequencing

Mice at the age of 3 months were sacrificed by perfusing saline after anesthesia. Brains were quickly removed and snap-frozen in liquid nitrogen to inactivate RNases before being sent to the Beijing Genomics Institute (BGI, China) for analysis. The brain samples were homogenized for RNA extraction according to the standard TRIzol protocol. RNAs were purified and fragmented before the reverse-transcription procedure to acquire the one-to-one corresponding cDNAs. cDNAs sequentially underwent end repair, PolyA affixion, and adapter assembly before being amplified by regular PCR. Amplified cDNAs were compared with the standard gene library for quality test and finally, loaded into the BGISEQ sequencer for RNA sequencing (mean reads length: PE150). The depth of the reads is not a valid reference in this kind of sequencing method. Instead, it is replaced by another similar concept, the amount of data. Each sample produced approximately 6.61GB amount of data.

### Physical Assessment and Behavioral Phenotyping

WT mice and age-matched N103 mice were weighed and tested for assessing their motor and non-motor functions by performing tail suspension test, open field test, rotarod test, grid performance test, pole test, and myodynamia measuring. In the tail suspension test, the experiment was performed as previously reported (Zhang et al., 2018). Mice were suspended by hanging tails in front of a blank background and monitored for at most 2 min. Pictures were taken when they were immobile. In the rotarod test, mice were placed on a spinning rod of a gradually accelerating spinning rate from 5 to 40 r.p.m. within 3 min. Mice were trained 3 times a day for 3 consecutive days before being subjected to the formal experiment. A 30-min break was given between trial intervals. In the grid performance test, mice were placed on a grid (1.5 m × 1.5 m with apertures of 5 mm diameter), which would be inverted after horizontally wobbled several times. Once the grid was flipped, mice were hanging upside down by their paws until they dropped onto the ground. The latency to fall was recorded to evaluate their endurance. In the open field test, mice were put in the middle of an open field (50 cm × 50 cm) and allowed to freely explore for 3 min. A monitoring camera was utilized for recording. 75% ethanol was applied to eliminate the odor and excrement in the apparatus between testing lags. Video data were analyzed with the ANY-maze behavioral tracking software (version 5.2). In the pole test, mice were placed head-upward on the top of a vertical rough-surfaced wooden pole (45 cm long, 1 cm diameter) and waited for autonomous descending. The formal test started after mice were subjected to three trials for training. Three parameters were measured: The hesitating latency before the mice oriented downward and started to go down; The interval between when mice started to descend and when reached the midpoint of the pole; The interval between when they arrived at the middle and when their paws touched the floor.

### Myodynamia Measuring

To measure the muscle strength of the forelegs, mice were prompted to horizontally grip the hook of a spring-loaded thrust meter and slowly pulled by their tails until they cannot insist. Maximal readings were recorded.

### Fecal Analysis

Approximately 0.5 g excrement of each mouse was collected and weighed before and after dried at 65°C for 2 h. A longer drying time won’t lead to further weight loss, suggesting the water had been fully depleted. The excrement water content of each mouse was obtained by calculating the ratio of the dry weight and the wet weight of the excrement, i.e., water content ratio% = dry weight (g)/wet weight (g).

### Statistical Analysis

All data were expressed as means ± s.e.m. from three or more independent experiments and illustrated with the GraphPad Prism (version 8.0). An unpaired student t-test was used to analyze the difference between two unpaired groups. One-way ANOVA was applied to confirm the significant main effects and differences among three or more groups followed by Tukey’s multiple comparisons for *post hoc* tests. For the time-course study, the level of significance was determined using the two-way ANOVA and Bonferroni’s multiple comparisons. Mann–Whitney *U* test was used to analyze the ratios between the two groups. Statistical significance was set as *P* < 0.05. The effect size of the samples was determined by Power analysis (Biostat).

## Results

### Generation of Transgenic Mice That Overexpress Human α-Synuclein 1-103 Fragment

**FIGURE 1:**
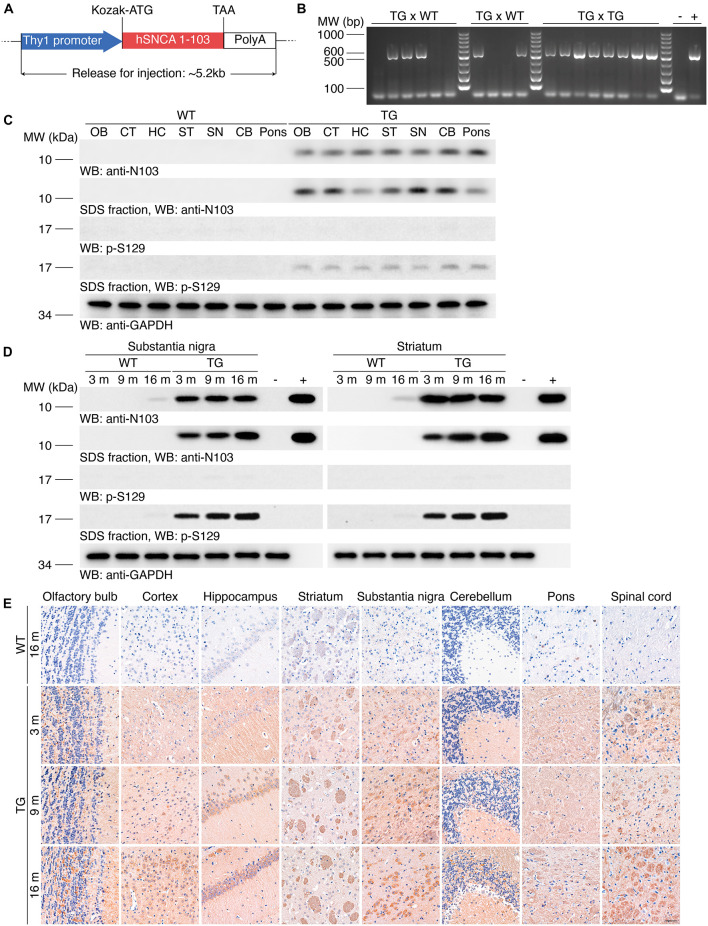
Generation of the N103 mouse line. **(A)** Schematic of the imported Thy1-α-synuclein 1-103 sequence. Kozak sequence (GCCGCCACC) was added before the start codon ATG for the ribosome to recognize the initiation codon. **(B)** Genotyping of the N103 mice. DNA samples of WT mice and N103 founder mice were loaded as negative control and positive control, respectively. **(C)** Western blots showing the expression of the α-synuclein 1-103 and α-synuclein p-S129 in soluble and insoluble fractions within various regions of the brains of the 6-month-old WT mice and N103 mice. GAPDH was used as the loading control in the soluble fractions. OB, olfactory bulb; CT, cortex; HC, hippocampus; ST, striatum; SN, substantia nigra; CB, cerebellum. **(D)** Western blots showing the soluble and insoluble α-synuclein 1-103 and α-synuclein p-S129 in the substantia nigra and striatum of the mouse brains. Brain lysates of 2-months-old WT mice and recombinant human α-synuclein 1-103 fragment were used as negative control and positive control, respectively. **(E)** Immunohistochemistry of α-synuclein 1-103 in different areas of the central nervous system in WT mice and N103 mice. Scale bar, 40 μm.

To generate transgenic mice that express human α-synuclein 1-103 fragments, we cloned the Thy1-α-synuclein 1-103 cDNAs, which drives the expression of the transgene in neurons (Figure 1A). These prepared sequences were imported into C57BL/6J mice. We consecutively crossbred the N103 mice with WT mice for at least three generations. Genotyping on the offspring suggests that the hereditary feature of this mouse line complies with the Mendelian law of inheritance, indicating the hereditary property of the N103 mouse line came to a stable state (Figure 1B). To verify the expression of the imported α-synuclein 1-103 gene, Western blots were performed using the brain lysates from different regions of WT mice and N103 mice. α-Synuclein 1-103 fragment was not detected in the brains of 6-months-old WT mice, but detectable in the age-matched N103 mice. We also tested the deposition of α-synuclein in the mice brain and found that the insoluble α-synuclein 1-103 fragment was particularly enriched in the classical PD-related regions including the olfactory bulb, cortex, striatum, substantia nigra, and pons (Figure 1C). Insoluble α-synuclein phosphorylated at Ser129 (α-synuclein p-S129) appeared within all areas of the central nervous system in N103 mice but not in age-matched control mice (Figure 1C). We further tested the age-dependent aggregation of α-synuclein in the substantia nigra and striatum and found that the insoluble α-synuclein 1-103 and α-synuclein p-S129 were accumulated in an age-dependent manner in N103 mice, while only very few soluble α-synuclein 1-103 and insoluble α-synuclein p-S129 were detected in the aged WT mice (Figure 1D, see *F* and *P* values in Supplementary Figure 1). To figure out the relative ratio of endogenous mouse α-synuclein and imported α-synuclein 1-103 in N103 mice. Brains of the 9-month-old WT mice and N103 mice were homogenized for Western blots using the anti-α-synuclein N-terminal antibody (Syn-1/Clone42). The content of α-synuclein 1-103 was approximately 1.6 times the endogenous mouse α-synuclein (see *F* and *P* values in Supplementary Figure 2).

**FIGURE 2:**
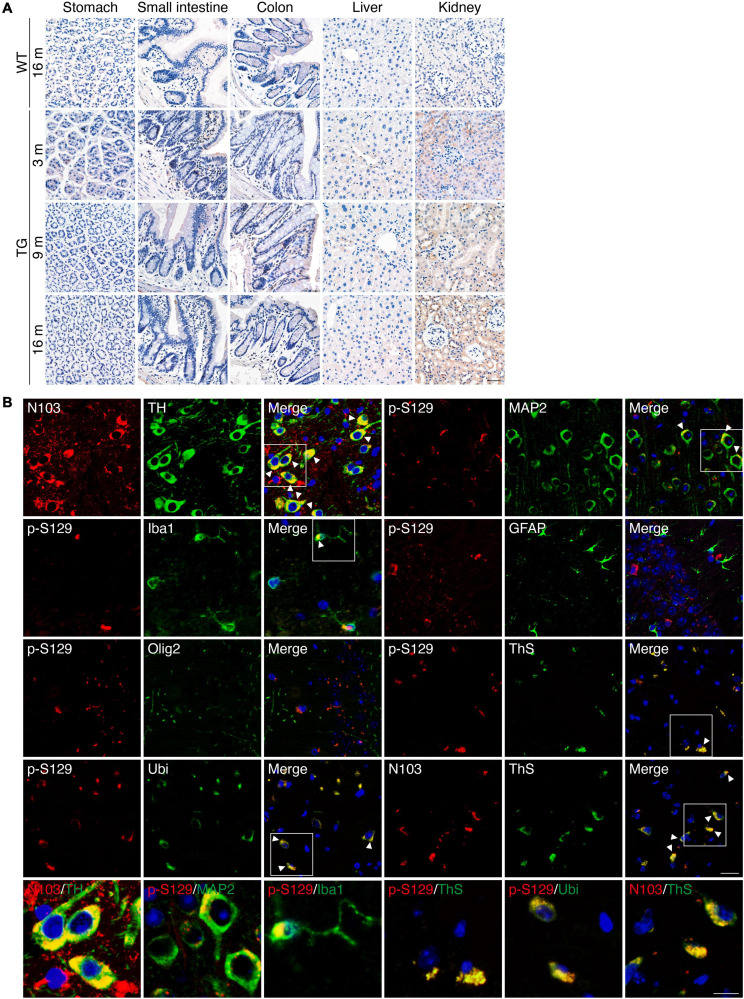
PD-like pathology in N103 mice. **(A)** Immunohistochemistry of α-synuclein 1-103 fragment in non-neural tissues of WT mice and N103 mice. Scale bar, 40 μm. **(B)** Double-label immunofluorescence of the substantia nigra, cortex, and hippocampus sections of 16-month-old N103 mice. α-synuclein 1-103 (red) and TH (green); α-synuclein p-S129 (red) and MAP2, Iba1, GFAP, Olig2, Ubiquitin, thioflavin-S (green); α-synuclein 1-103 (red) and thioflavin-S (green). Scale bar, 20 μm. Colocalization is marked with white arrowheads and zoomed below. Scale bar, 10 μm.

The expression of α-synuclein 1-103 fragment in different brain regions of the N103 mice was analyzed using immunohistochemistry. The α-synuclein 1-103 was undetectable in 3-month-old WT mice and rare in the brain of 16-month-old WT mice, but abundantly expressed in an age-dependent manner in the N103 mice, especially in the mitral cell layer and granule cell layer of the olfactory bulb, pyramidal neurons in the outer granular layer of the cortex, the CA1, CA2 areas and dentate gyrus of the hippocampus, the substantia nigra, and the molecular layer of the cerebellum. It was also detected in the white matter bundles traveling through the striatum and spinal cord (Figure 1E, see *F* and *P* values in Supplementary Figures 3, 4A, 5). We also investigated the expression of α-synuclein 1-103 fragments in the non-neural organs by immunohistochemistry analysis. The α-synuclein 1-103 fragment was barely detected within these tissues except the kidney (Figure 2A and Supplementary Figure 6A), where a large amount of α-synuclein 1-103 appeared in the epithelial cells of convoluted tubules and less in the loops of Henle (Supplementary Figure 6B).

Immunofluorescence with anti-α-synuclein 1-103 and anti-tyrosine hydroxylase (TH) antibodies using sections of the substantia nigra of the N103 mice confirmed that α-synuclein 1-103 was abundantly expressed in the TH-positive dopaminergic neurons (Figure 2B). Interestingly, α-synuclein 1-103 appeared in a form of more compact chunks in the TH-positive cells of the substantia nigra rather than small round pieces in the TH-negative cells in the rostral interstitial nucleus and other regions (see *t* and *P* values in Supplementary Figure 7). To investigate the type of cells that express pathological α-synuclein, immunofluorescence analysis on the coronal sections through the planes of the substantia nigra of the 9-months-old N103 mice was performed. It showed that MAP2-positive neurons in the cortex express α-synuclein p-S129. Only mild α-synuclein p-S129 appeared in the cytoplasm of microglia, which might be a consequence of α-synuclein p-S129 being absorbed by microglia. There was no visible colocalization of α-synuclein p-S129 and GFAP as well as Olig2, suggesting that α-synucleinopathy does not jeopardize astrocytes and oligodendrocytes in this mouse line. Moreover, α-synuclein p-S129 and α-synuclein 1-103 in the cortex are positive for thioflavin-S staining, suggesting that the α-synuclein 1-103 fragment forms amyloid structures. Previous studies reported that the proteins in the LBs are highly ubiquitinated, and dysfunction of the ubiquitin-proteasome system is involved in PD-associated pathology (McNaught et al., 2001; McKinnon et al., 2020). To figure out if this happened in N103 mice, we stained the brain slides with an anti-ubiquitin antibody and found that α-synuclein p-S129 colocalize with ubiquitin (Figure 2B). To confirm the aggregation of α-synuclein, we stained the brain sections of WT mice and N103 mice using an anti-α-synuclein aggregate antibody (MJFR 14-6-4-2). More compact α-synuclein aggregates were detected in the olfactory bulb, cortex, striatum, substantia nigra, hippocampus, cerebellum, and pons of the aged N103 mice when compared with the younger N103 mice and the age-matched control (Supplementary Figure 8). A similar pathological feature was detected in the spinal anterior horn. Abundant α-synuclein aggregates were detected in the anterior horn of the spinal cord. Granular aggregates appeared in motor neurons of the N103 mice, but not in those of WT mice (see *t* and *P* values in Supplementary Figures 4B,C). Collectively, the N103 mouse line steadily expressed the imported α-synuclein 1-103 gene and displayed progressive pathological protein deposits in the central nervous system.

### N103 Mice Show Early α-Synucleinopathy and Progressive Degeneration of the Nigrostriatal Pathway

**FIGURE 3:**
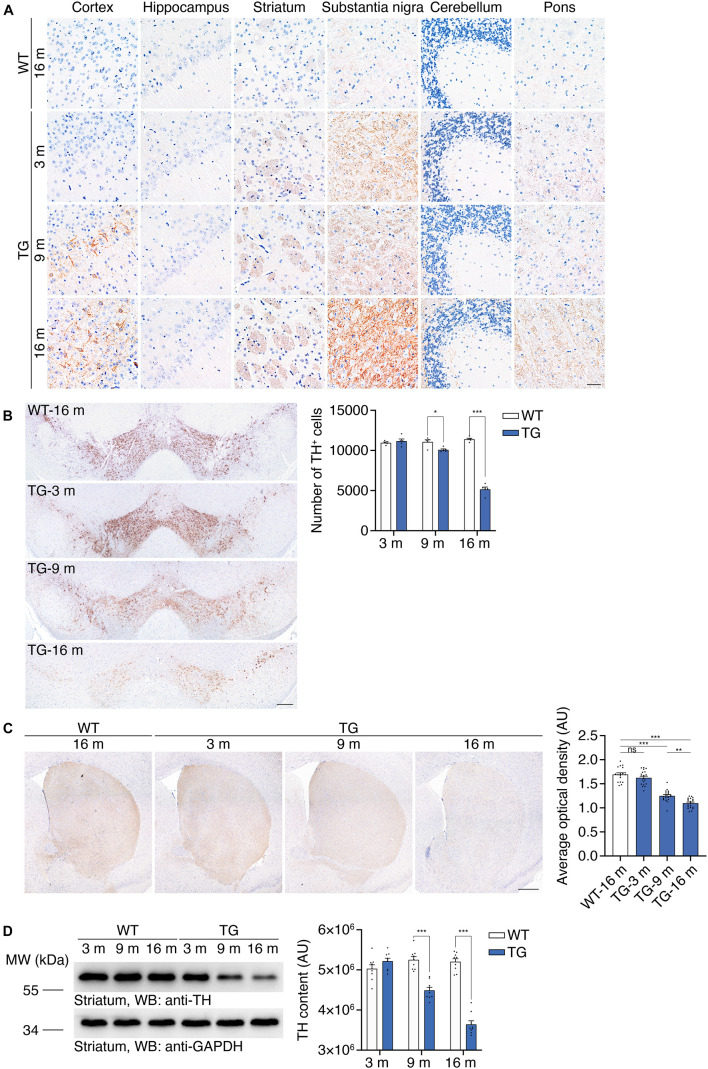
Accumulation of α-synuclein p-S129 and nigrostriatal pathway degeneration in N103 mice. **(A)** Immunohistochemistry of α-synuclein p-S129 in the cortex, hippocampus, striatum, substantia nigra, cerebellum, and pons in WT mice and N103 mice. Scale bar, 40 μm. **(B)** Unbiased stereological cell counting of the substantia nigra by anti-TH immunostaining. Scale bars, 200 μm. Bar graph, quantification of the TH-positive neurons. Data are mean ± s.e.m.; *n* = 5 mice per group; *P < 0.05 and ***P < 0.001 by two-way ANOVA and Bonferroni’s multiple comparisons. **(C)** TH immunostaining of striatum. Scale bars, 400 μm. Bar graph, quantification of the average optical density (expressed as arbitrary units). Data are mean ± s.e.m.; *n* = 5 mice per group; **P < 0.01 and ***P < 0.001 by one-way ANOVA and Tukey’s multiple comparisons. **(D)** Western blots showing the striatal TH content in WT mice and N103 mice. Bar graph, quantification of the integrated optical intensity (expressed as arbitrary units). Data are shown as mean ± s.e.m.; *n* = 9 mice per group; ***P < 0.001 by two-way ANOVA and Bonferroni’s multiple comparisons.

Hyperphosphorylation and aggregation of α-synuclein is a characteristic pathological feature in the brains of PD patients. The α-synuclein aggregates propagate through a stereotypical pathway, known as the Braak staging (Braak et al., 2003). We tested the age-dependent propagation of α-synucleinopathy in the N103 mouse line to illustrate its propagation pattern through the central nervous system. Immunohistochemistry was conducted with the brain sections of 16-month-old WT mice and N103 mice at 3, 9, 16 months. We found that α-synuclein p-S129 emerged at the age of 3 months. The substantia nigra, striatum, and pons were the first regions to be involved. There was very slight α-synuclein pathology found in the cerebellum at the age of 3 months. Cortex was affected at 9 months and turned to be a very part where a large amount of compact α-synuclein aggregations emerged. It is worth noting that it’s α-synuclein p-S129-positive neurites instead of the classical LB-like pathology that constituted the major pathological feature in the substantia nigra and reticular formation nuclei of the pons (Figure 3A, see *F* and *P* values in Supplementary Figure 9). Similar results were confirmed by the protease K digestion assay in the olfactory bulb, cortex, striatum, substantia nigra, hippocampus, cerebellum, and pons (see *t* and *P* values in Supplementary Figure 10). Immunohistochemistry of the spinal cord with anti-α-synuclein p-S129 antibody after protease K digestion suggested that fine granular α-synuclein p-S129 accumulated in the spinal cord (see *t* and *P* values in Supplementary Figures 4D,E). However, typical LBs were missing from the cytoplasm of the motor neurons in the anterior horn (Supplementary Figure 4D). In conclusion, α-synucleinopathy in the N103 mice develops in a way similar to that of the PD patients.

We further investigated whether the N103 mice mimic the loss of dopaminergic neurons in the substantia nigra. Stereological counting of the dopaminergic neurons in the substantia nigra was performed using consecutive sections of WT mice and N103 mice of different ages. Significant TH-positive cell loss was observed in the N103 mice after 9 months but not at the age of 3 months (F_Time_ = 112.4, F_Group_ = 148.8, *p* < 0.001) (Figure 3B). Immunohistochemistry was conducted on 16-months-old WT mice and N103 mice at different ages to quantify the volume of TH-positive dopaminergic projection in the striatum. The staining signal was remarkably faint in the sections of aged N103 mice when compared with the age-matched WT control (one-way ANOVA followed by Tukey’s multiple comparisons test, *F* = 96.13, *p* < 0.001) (Figure 3C). This result was confirmed by Western blots of the striatal TH (F_Time_ = 33.24, F_Group_ = 149.4, *p* < 0.001) (Figure 3D). In conclusion, the N103 mice mimic two major characteristic pathologies of human PD: the pathological α-synuclein aggregation that propagated following a pattern similar to that in sporadic PD, and age-dependent progressive nigrostriatal dopaminergic degeneration.

### Age-Dependent Striatal Synaptic Degeneration in N103 Mice

**FIGURE 4:**
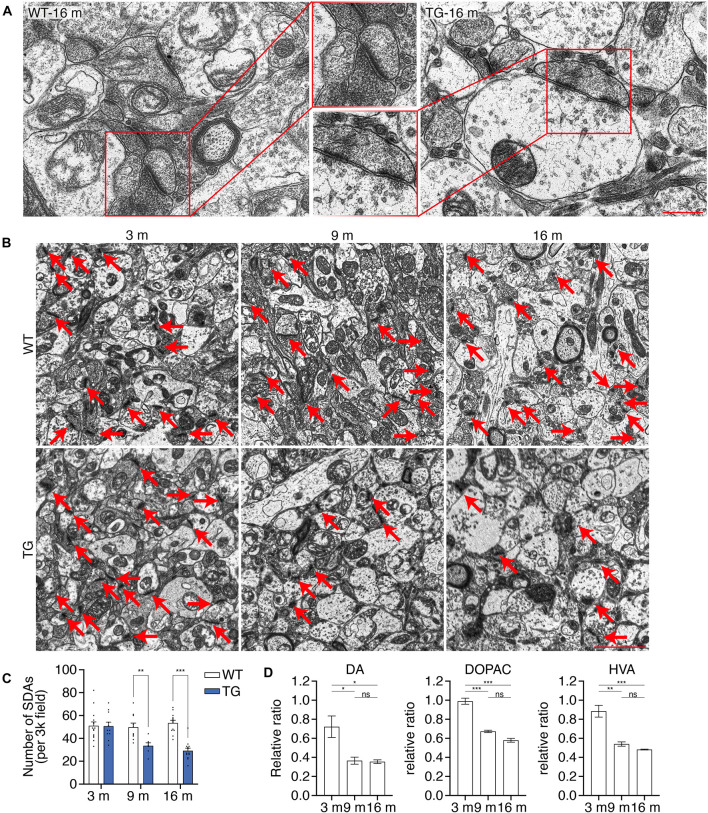
N103 mice demonstrate significant striatal synaptic degeneration. **(A)** Representative TEM images of the striatal synaptic vesicles in the aged WT control and N103 mice. Scale bar, 0.5 μm. Regions of interest were zoomed. **(B)** TEM images of the striatal synapses. Red arrows indicate the synaptic dense areas (SDAs). Scale bar, 2 μm. **(C)** Bar graph, quantification of the number of SDAs per 3k field. Data are mean ± s.e.m.; images from *n* = 3 mice per group; **P < 0.01 and ***P < 0.001 by two-way ANOVA and Bonferroni’s multiple comparisons. **(D)** HPLC analysis of the striatal neurotransmitters (DA, DOPAC, and HVA) in N103 mice and WT littermates. Data are mean ± s.e.m.; samples from *n* = 3 mice per group; *P < 0.05, **P < 0.01, ***P < 0.001 by Mann–Whitney *U* test.

Degeneration of dopaminergic terminals in the striatum is found in PD patients (Reeve et al., 2018; Nguyen et al., 2019). To illustrate a detailed description of the synaptic dysfunction in the nigrostriatal pathway, transmission electron microscopy (TEM) analysis was performed using the ultrathin sections of the striatum of WT mice and N103 mice at different ages. Representative pictures showed affluent vesicles in the post synapses of 16-months-old WT mice while only sparse ones were found in aged N103 mice (Figure 4A). Quantification of the number of synaptic density areas (SDAs) in N103 mice displayed a significant reduction compared with the age-matched controls (F_Time_ = 6.969, *P* = 0.0044, F_Group_ = 24.71, *P* < 0.001) (Figures 4B,C). We also quantified the concentration of dopamine and its metabolites DOPAC and HVA in the striatum using high-performance liquid chromatography (HPLC). The concentration of dopamine, DOPAC, and HVA was rapidly decreased at the ninth month of their life span (Figure 4D, see *F* and *P* values in Supplementary Figure 11). These results indicate that nigrostriatal pathway impairment was rooted in the structural damage of the dopaminergic neurons’ far-end terminals.

### Motor and Non-motor Symptoms of N103 Mice

**FIGURE 5:**
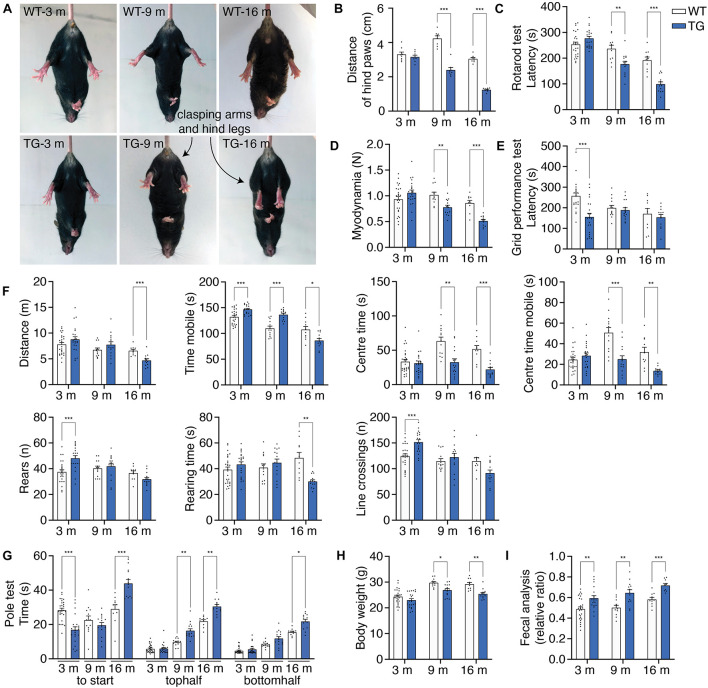
N103 mice show behavioral impairment, weight loss, and constipation. **(A)** Representative images of tail suspension test presenting clasping arms and legs of N103 mice. **(B)** Quantification analysis of the mean distances of the hind paws of WT and N103 mice in **(A)**. Data are mean ± s.e.m.; *n* = 8 mice per group. **(C)** Rotarod test of WT and N103 mice. **(D)** Myodynamia test of WT mice and N103 mice. **(E)** Grid performance test of WT littermates and N103 mice. **(F)** Open field test assessing the locomotion behavior of control group and N103 mice. **(G)** Pole test revealing the exploration intention and motor function of WT mice and N103 mice. **(H)** Bodyweight of WT mice and N103 mice at different ages. **(I)** Fecal analysis showing the excremental water content of the control and N103 mice. **P < 0.01 and ***P < 0.001 by Mann–Whitney *U* test. **(C–I)** Data are mean ± s.e.m.; *n* = 27 WT mice of 3 months, *n* = 13 WT mice of 9 months, *n* = 10 WT mice of 16 months, *n* = 22 N103 mice of 3 months, *n* = 15 N103 mice of 9 months, and *n* = 13 N103 mice of 16 months. **(B–H)** *P < 0.05, **P < 0.01, and ***P < 0.001 by two-way ANOVA and Bonferroni’s multiple comparisons.

WT mice and N103 mice at 3, 9, and 16 months were involved in behavioral tests to assess their locomotion activity, muscle strength, stamina. In the tail suspension test, aged N103 mice showed clasping legs rather than spreading out to both sides of their bodies as the WT mice. Mean distances of hind paws were declined at the age of 9 months (4.233 vs. 2.400 cm) and 16 months (1.245 vs. 3.945 cm) (F_Time_ = 79.48, F_Group_ = 129.4, *p* < 0.001) (Figures 5A,B). Aged N103 mice have worse performance in the rotarod test (F_Time_ = 65.95, F_Group_ = 23.02, *p* < 0.001) and myodynamia measuring (F_Time_ = 16.42, F_Group_ = 9.379, *p* < 0.001), suggesting their muscle strength declined after the age of 9 months (Figures 5C,D). In the grid performance test, 3-month-old N103 mice have a shorter latency to fall. Interestingly, this phenomenon did not occur in N103 mice of elder age. This might be caused by the fact that the 3-months-old N103 mice have a stronger tendency to spontaneously jump off due to anxiety (F_Time_ = 3.136, *p* = 0.0603, F_Group_ = 6.929, *p* = 0.0115) (Figure 5E), which is consistent with the results of the open field test (Figure 5F) and pole test (Figure 5G). In the open field test, N103 mice of 16 months move less (F_Time_ = 14.62, *p* < 0.001, F_Group_ = 0.004810, *p* = 0.9449) and spent more time in the peripheral area near the wall. The residence time (F_Time_ = 7.485, *p* = 0.0016, F_Group_ = 33.03, *p* < 0.001) and mobile time (F_Time_ = 10.96, F_Group_ = 26.09, *p* < 0.001) in the center started to decline at the age of 9 months. However, young N103 mice cross more lines and have more active time, which decreased at 16 months (F_Time_ = 20.09, *p* < 0.001, F_Group_ = 0.6921, *p* = 0.4097). N103 mice reared more at the age of 3 months but have less rearing time at the age of 16 months (F_Time_ = 9.034, *p* < 0.001, F_Group_ = 2.168, *p* = 0.1444) (Figure 5F). In the pole test, young N103 mice spent less time hesitating and take action earlier to descend compared with the age-matched WT mice (F_Time_ = 21.29, F_Group_ = 222.7, *p* < 0.001). However, this difference reversed at the age of 16 months, suggesting that aged N103 mice have a remarkably longer latency to climb down. When comparing the time for descending, elder N103 mice spent more time reaching the middle point (F_Time_ = 5.156, *p* = 0.0293, F_Group_ = 33.03, *p* < 0.001) and down to the ground (F_Time_ = 54.73, F_Group_ = 58.56, *p* < 0.001) (Figure 5G). Physical examination showed that N103 mice were lighter than the WT mice after the age of 9 months. This trend even expanded when the age reaching 16 months (F_Time_ = 34.70, F_Group_ = 21.38, *p* < 0.001) (Figure 5H). PD patients usually suffer from constipation, which is considered to be a classical non-motor symptom that happened much earlier than the motor symptoms. To investigate if a similar phenomenon occurred in this N103 mouse line, the water content of the excrement was calculated. The N103 mice excrete drier stool from a very early age (Figure 5I). When analyzed by gender, N103 mice did not manifest a gender-associated phenotype at different ages, suggesting that transgene was not inserted into sex chromosomes (see *F* and *P* values in Supplementary Figure 12). In conclusion, the N103 mice develop PD-like motor and non-motor symptoms in an age-dependent manner.

### Transcriptomics Pattern Was Extensively Altered in N103 Mice

**FIGURE 6:**
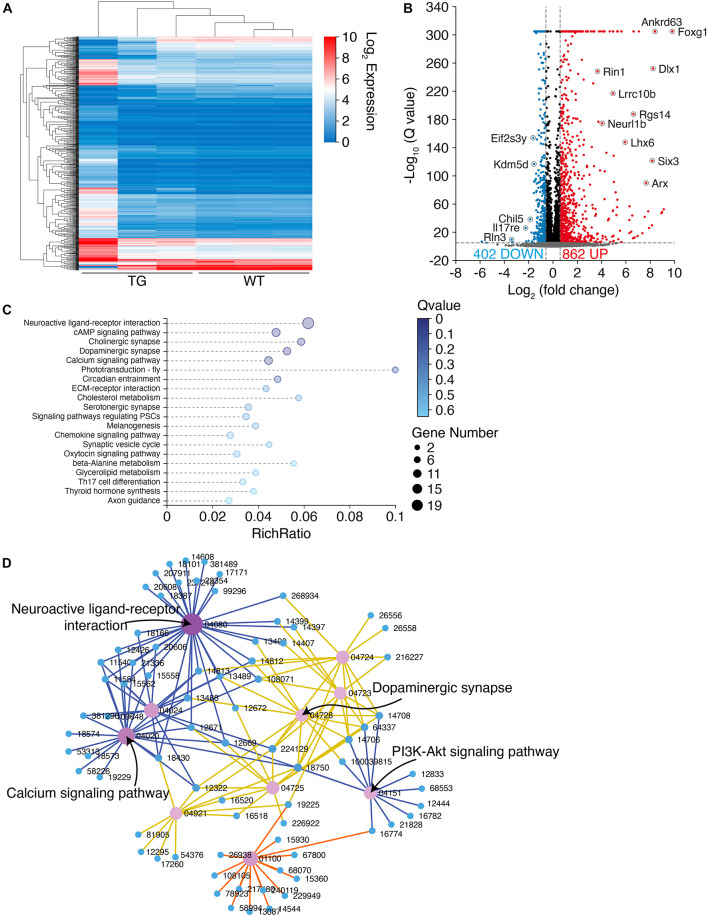
RNA-sequencing illustrates an extensive transcriptome change in N103 mice. **(A)** Clustering heat map giving an overview of the altered transcriptomics of WT mice and N103 mice. *n* = 3 mice per group. **(B)** Volcano plot revealing the differentially expressed genes (DEGs) of the control group and N103 mice. Downregulated DEGs (blue) and upregulated DEGs (red) were filtered out by a threshold set as –log_10_ Q value > 5 and | fold change (FC)| > 1.5. Most significant genes were annotated. **(C)** Enrichment analysis displaying a list of KEGG pathway terms gathering most DEGs. **(D)** KEGG interaction network illustrating the relationship among the most significant DEGs ranked by the Q values.

To explore the molecular mechanisms underlying the PD-like pathology in N103 mice, we performed RNA-sequencing analysis of N103 mice and WT controls at the age of 3 months. The clustering heat map provided an overview of the distinct expression patterns between these two groups (Figure 6A). By setting an appropriate screening threshold (−log_10_ Q value > 5 and | fold change (FC)| > 1.5), 1,246 differentially expressed genes (DEGs) were filtered out from 67,229 genes read by whole transcriptome resequencing. Among them, 862 genes were upregulated while the others were downregulated (Figure 6B). To figure out how the DEGs were engaged in the biological process of N103 mice, the KEGG-based enrichment analysis was performed. The most significant assembly was neuroactive ligand-receptor interaction with a rich ratio of 0.06. The other terms including cAMP signaling pathway, cholinergic synapse, dopaminergic synapse, and axon guidance appeared as well (Figure 6C). Next, we built a KEGG interaction network to illustrate the relationships of all DEGs. After eliminating isolated genes that did not interplay with the other DEGs, residuals were connected into several key nodes: neuroactive ligand–receptor interaction, calcium signaling pathway, PI3K-Akt signaling pathway (Figure 6D). In conclusion, the N103 mouse line experienced extensive alteration of RNA expression modes from an early age.

## Discussion

Here we reported a brand-new mouse line expressing the human α-synuclein 1-103 fragment. α-Synuclein 1-103 is abundant in the central nervous system but rare in the other organs except for the kidney where a lot of α-synuclein 1-103 fragments were detected in the renal tubular epithelial cells. By elaborate characterization, this line demonstrates various progressive PD pathological features, such as the accumulation of α-synuclein aggregates in PD-associated regions, loss of dopaminergic neurons and neurites in the nigrostriatal circuit. The generation of α-synuclein p-S129 could be ascribed to the aggregated α-synuclein 1-103 fragment, which acts as a seed to induce the deposition of endogenously expressed mouse α-synuclein. Furthermore, N103 mice well-replicated some non-motor symptoms of PD patients including weight loss and constipation. In the behavioral test, significant motor dysfunctions appeared during aging in N103 mice. Locomotion disability and anxiety were also observed.

Neurons perform their physiological functions by properly releasing neurotransmitters through a reliable synaptic structure. Toxic protein aggregates may cause damage to the neurites and synapses before the formation of cytoplasmic inclusions. Interestingly, α-synuclein 1-103 has a stronger tendency to form compact chunks in the TH-positive cells of the substantia nigra rather than small round pieces in the TH-negative cells (Supplementary Figure 7). These results imply that the protein recycling process has been even suppressed due to the tremendous neurotoxicity of α-synuclein 1-103 fragments in the TH-positive dopaminergic neurons. In other words, TH-positive dopaminergic neurons may have some unknown vulnerabilities that significantly enhanced the toxicity of α-synuclein 1-103. From the perspective of synapsis plasticity, healthy synaptic structures are the precondition for the neurons to perform their physiological functions and well-functioning synapses protect neurons from apoptosis. Further studies are needed to elucidate these vulnerabilities and the underlying synaptic mechanisms of PD.

A PD mouse model overexpressing wild-type human α-synuclein under Thy1 promoter (Thy1-α-synuclein mice) was established by Fleming (2004). Compared with this model, N103 mice demonstrate significant weight loss after the age of 9 months while that of the Thy1-α-synuclein mice starts to decline before the age of 2 months. N103 mice rear more when compared with the age-matched 3-month-old WT mice and, conversely, the age-matched Thy1-α-synuclein mice rear less.

Some mouse lines expressing truncated α-synuclein have been constructed and characterized in previous studies (Sorrentino and Giasson, 2020). Tofaris et al. constructed the α-synuclein 1-120 transgenic mouse driven by TH promoter on a mouse α-synuclein null background. Pathological inclusions in the substantia nigra and olfactory bulb have been detected (Tofaris, 2006). These results indicate that certain truncated α-synuclein fragments could induce PD-like pathology in the absence of endogenous α-synuclein. α-Synuclein 1-130 transgenic mouse established by Wakamatsu et al. showed a selective neuronal loss in the substantia nigra, striatal synaptic impairment, and reduced DA content in the striatum. The mice showed reduced locomotor activity, which was relieved by levodopa treatment. However, unlike what happens in the PD brain, the dopaminergic neurodegeneration of this mouse model was not age-dependent and seemed to happen during embryogenesis (Wakamatsu et al., 2008a,b). α-Synuclein 1-119 fragments have been found to be present in the PD brains (Li et al., 2005; Anderson et al., 2006; Lewis et al., 2010; Prasad et al., 2012; Kellie et al., 2015). Daher et al. designed α-synuclein 1-119 mice under the control of endogenous murine ROSA26 promoter in a Cre recombinase-dependent manner. This model manifested decreased striatal DA level but failed to induce dopaminergic neuronal loss by 12 months (Daher et al., 2009).

Although several mouse models expressing truncated α-synuclein have been constructed, the N103 mouse model is unique in some respects. Firstly, α-synuclein aggregates and deposits in this model follow a similar pattern as the Braak staging in human cases. It is interesting that the aggregation of α-synuclein is rather prominent in the substantia nigra, which is different from other Thy1-based models (Kahle et al., 2000; van der Putten et al., 2000; Chesselet et al., 2012). Secondly, compared with the well-recognized α-synuclein A53T transgenic mice, which express full-length A53T mutant α-synuclein, N103 mice show reproducible PD pathology under a lower expressing load (4.6-fold versus 2.6-fold of transgene expression over endogenous α-synuclein). Even if under a lower expressing load, the N103 model shows α-synuclein inclusions as early as 3 months, while the earliest pathological changes start to emerge at the age of 7 months in α-synuclein A53T transgenic mice (Giasson et al., 2002). Striatal synaptic degeneration is also observed in N103 mice at an early age. Thirdly, it is worth noting that N103 mice not only developed PD-like motor symptoms, but also developed non-motor symptoms including weight loss, constipation, and anxiety. This is very important since some of the non-motor symptoms happen earlier than the motor symptoms, significantly influence the quality of life, and determine the prognosis of PD.

In addition, compared with other models expressing truncated α-synuclein, N103 mice were designed to develop PD-like pathology through a common molecular mechanism that has been proved to extensively exists in human sporadic PD. Our previous studies have proved that AEP is activated in an age-dependent manner, which is consistent with the age-dependent onset of PD. The AEP-derived α-synuclein 1-103 fragment is extensively present in the brains of patients with sporadic PD, suggesting that the production of α-synuclein 1-103 is a general biological event in sporadic PD (Zhang et al., 2017a). A mouse model based on such molecular mechanisms may closely mimic what is happening in the real world.

N103 mice might become a powerful tool for PD research. However, this is a transgenic mouse model that overexpresses α-synuclein 1-103 fragments in the neurons. The level of α-synuclein 1-103 fragment in this model is higher than that in the brain of PD patients. Thus, this model may not exactly mimic the pathogenesis of PD. Furthermore, by which mechanism the α-synuclein 1-103 fragments promote the development of PD pathology have not been clearly clarified in this study, although some clues raised by the N103 mice point to the synaptic impairment and neuronal receptor disorder. α-Synuclein 1-103 fragment may damage neurons by binding to synaptic proteins or the energy supply-associated molecules and organelles, which needs to be elucidated in the future.

## Conclusion

We established a brand-new transgenic mouse line expressing human α-synuclein 1-103 fragments. By elaborate characterization, it showed classical PD-like pathologies in an age-dependent manner, such as the accumulation of pathological α-synuclein in the central nervous system, especially in the PD-associated regions, and the significant nigrostriatal degeneration. Motor impairments and some non-motor symptoms also appeared in this mouse line. The RNA-sequencing analysis found that the transcriptomics pattern was extensively altered in N103 mice. The results suggest that the N103 mice may serve as a novel model that mimics the onset of sporadic PD.

## Data Availability Statement

The original contributions presented in the study are included in the article/Supplementary Material, further inquiries can be directed to the corresponding author/s.

## Ethics Statement

The animal study was reviewed and approved by the Animal Ethics Committee of the Renmin Hospital of Wuhan University.

## Author Contributions

YT performed most of the experiments, analyzed the data, and wrote the draft. LP, XY, MX, and ZYu helped in designing the methodology. MH and LM participated in resource management. ZYa conducted the formal analysis. KY helped in designing the experiments. ZZ conceived the project and designed the experiments. All authors contributed to the article and approved the submitted version.

## Conflict of Interest

The authors declare that the research was conducted in the absence of any commercial or financial relationships that could be construed as a potential conflict of interest.

## Publisher’s Note

All claims expressed in this article are solely those of the authors and do not necessarily represent those of their affiliated organizations, or those of the publisher, the editors and the reviewers. Any product that may be evaluated in this article, or claim that may be made by its manufacturer, is not guaranteed or endorsed by the publisher.

## Supplementary Material

Supplementary Figure 1 Quantification of Western blots showing the soluble and insoluble α-synuclein 1-103 and α-synuclein p-S129 in the substantia nigra and striatum of the mouse brains (expressed as arbitrary units). SN, substantia nigra; ST, striatum. Data are shown as mean ± s.e.m.; *n* = 4 mice per group; ^∗∗∗^*P* < 0.001 by two-way ANOVA and Bonferroni’s multiple comparisons. F and *P* values are: F_SN–N103 Time_ = 8.389, *P* = 0.0151, F_SN–N103 Group_ = 11128, *P* < 0.001; F_SN–N103(SDS) Time_ = 35.69, *P* < 0.001, F_SN–N103(SDS) Group_ = 3181, *P* < 0.001; F_*SN–p–S129 Time*_ = 4.369, *P* = 0.0284, F_*SN–p–S129 Group*_ = 0.3896, *P* = 0.5403; F_SN–p–S129(SDS) Time_ = 31.10, *P* < 0.001, F_SN–p–S129(SDS) Group_ = 3307, *P* < 0.001; F_ST–N103 Time_ = 7.176, *P* = 0.0051, F_ST–N103 Group_ = 2528, *P* < 0.001; F_ST–N103(SDS) Time_ = 94.40, *P* < 0.001, F_ST–N103(SDS) Group_ = 2365, *P* < 0.001; F_ST–p–S129 Time_ = 0.2137, *P* = 0.8096, F_ST–p–S129 Group_ = 0.3859, *P* = 0.5423; and F_ST–p–S129(SDS) Time_ = 81.04, *P* < 0.001, F_ST–p–S129(SDS) Group_ = 4429, *P* < 0.001.

Supplementary Figure 2 Western blots showing the expression of the endogenous mouse α-synuclein and imported α-synuclein 1-103 in WT mice and N103 mice at the age of 9 months. Bar graph, quantification of the Western blot results (expressed as arbitrary units). Data are shown as mean ± s.e.m.; *n* = 6 mice per group; ^∗∗∗^*P* < 0.001 by two-way ANOVA and Bonferroni’s multiple comparisons. F and *P* values are: F_Protein_ = 2.335, *P* = 0.1422 and F_Group_ = 50.56, for all *P* < 0.001.

Supplementary Figure 3 Quantification of the IHC results showing α-synuclein 1-103 in the central nervous system of N103 mice (expressed as arbitrary units). Data are shown as mean ± s.e.m.; *n* = 6 slices per group; ns., non-specific, ^∗^*P* < 0.05, and ^∗∗^*P* < 0.01; all the unmarked comparisons among these groups mean *P* < 0.001 by one-way ANOVA and Tukey’s multiple comparisons. *F* and *P* values are: F_Olfactory bulb_ = 241.8, F_Cortex_ = 191.7, F_Hippocampus_ = 176.7, F_Striatum_ = 421.0, F_Substantia nigra_ = 237.2, F_Cerebellum_ = 337.2, F_Pons_ = 91.48, and F_Spinal cord_ = 345.9, for all *P* < 0.001.

Supplementary Figure 4 Immunohistochemistry and immunofluorescence analysis showing abundant pathological α-synuclein in the spinal cord of N103 mice. **(A)** α-Synuclein 1-103 fragments in the white matter bundles traveling through the dorsal horn of the spinal cord. Scale bar, 160 μm. Images are zoomed below. **(B)** α-Synuclein filaments labeled by MJFR 14-6-4-2 antibody in the spinal cord anterior horn of the aged N103 mice. Rare was detected in the age-matched WT mice. Motor neurons were outlined. Scale bar, 80 μm. **(C)** Quantification of the integrated fluorescence intensity (expressed as arbitrary fluorescence units) of **(B)**. Data are shown as mean ± s.e.m.; *n* = 6 slices per group; ^∗∗∗^*P* < 0.001 by unpaired student *t*-test (*t* = 44.26, *P* < 0.001). **(D)** Proteinase K digestion showing fine granular hydrolysis-resistant α-synuclein p-S129 in motor neurons in the spinal anterior horn of the aged N103 mice. **(E)** Quantification analysis of the integrated fluorescence intensity (expressed as arbitrary units) of **(D)**. Data are shown as mean ± s.e.m.; *n* = 8 slices per group; ^∗∗∗^*P* < 0.001 by unpaired student *t*-test (*t* = 33.46, *P* < 0.001).

Supplementary Figure 5 Expression of α-synuclein 1-103 fragment in different regions of the central nervous system in the WT mice at the age of 3 and 9 months. Brain sections of WT mice were stained with the anti-N103 antibody. Scale bar, 40 μm.

Supplementary Figure 6 The expression of α-synuclein 1-103 in the non-neural tissues of WT mice and N103 mice. **(A)** α-Synuclein 1-103 in the non-neural tissues of WT mice at different ages. Scale bar, 40 μm. **(B)** Panorama showing the distribution pattern of α-synuclein 1-103 in the kidney of 9-month-old N103 mice.

Supplementary Figure 7 α-Synuclein 1-103 is more prone to form compact chunks in the TH-positive cells of the substantia nigra rather than small round pieces in the TH-negative cells. Regions of interest are zoomed. Scale bar, 20 μm. Quantification showing diameter of N103 inclusions in the substantia nigra (SN) and a region near SN, rostral interstitial nucleus (RIN). Data are shown as mean ± s.e.m.; *n* = 25 slices for SN and *n* = 24 slices for RIN; ^∗∗∗^*P* < 0.001 by unpaired student *t*-test (4.589 μm vs. 2.000 μm, *t* = 9.384, *P* < 0.001).

Supplementary Figure 8 Abundant α-synuclein aggregates are detected in the brains of the aged N103 mice. Immunofluorescence using MJFR 14-6-4-2 antibody showing the α-synuclein filaments in different areas of the central nervous system in N103 mice at the age of 2 and 16 months, and 16-month-old WT mice. Scale bar, 80 μm. Regions of interest are zoomed in the middle. The positive staining area is masked by binarization with a proper brightness threshold for baseline correction. The outputs are illustrated below.

Supplementary Figure 9 Quantification analysis of the accumulated α-synuclein p-S129 in the central nervous system of N103 mice (expressed as arbitrary units). Data are shown as mean ± s.e.m.; *n* = 6 slices per group; ns., non-specific, ^∗∗^*P* < 0.01; all the unmarked comparisons among these groups mean *P* < 0.001 by one-way ANOVA and Tukey’s multiple comparisons. F and *P* values are: F_Cortex_ = 468.3, F_Hippocampus_ = 148.0, F_Striatum_ = 1085, F_Substantia nigra_ = 1266, F_Cerebellum_ = 418.6, and F_Pons_ = 583.9, for all *P* < 0.001.

Supplementary Figure 10 Proteinase K digestion showing hydrolysis-resistant α-synuclein p-S129 in the brains of 16-month-old WT mice and N103 mice. Digestion-resistant α-synuclein p-S129 is mainly detected in the cortex, substantia nigra, and pons. Scale bar, 80 μm. Quantification showing the integrated optical intensity (expressed as arbitrary units). Data are shown as mean ± s.e.m.; *n* = 8 slices per group; ^∗∗∗^*P* < 0.001 by unpaired student *t*-test (t_Olfactory bulb_ = 38.15, t_Cortex_ = 43.31, t_Hippocampus_ = 4.641, t_Striatum_ = 26.76, t_Substantia nigra_ = 47.93, t_Cerebellum_ = 29.96, and t_Pons_ = 34.70, for all *P* < 0.001).

Supplementary Figure 11 HPLC analysis of the striatal neurotransmitters (DA, DOPAC, and HVA) in N103 mice and WT littermates. Results were expressed as absolute contents (ng/mg). Data are mean ± s.e.m.; samples from *n* = 3 mice per group; ^∗^*P* < 0.05 and ^∗∗∗^*P* < 0.001 by two-way ANOVA and Bonferroni’s multiple comparisons (for DA, F_time_ = 35.56, F_group_ = 70.88; for DOPAC, F_time_ = 124.6, F_group_ = 210.1; for HVA, F_time_ = 70.38, and F_group_ = 185.7; for all *P* < 0.001).

Supplementary Figure 12 Behavioral impairment and constipation of N103 mice do not demonstrate gender differences. Open field test (for distance, F_time_ = 8.454, *P* = 0.0009, F_group_ = 1.922, *P* = 0.1726; for time mobile, F_time_ = 20.27, *P* < 0.001, F_group_ = 0.06189, *P* = 0.8061; for line crossings, F_time_ = 9.005, *P* = 0.0006, F_group_ = 1.436, *P* = 0.2372; for rears, F_time_ = 5.877, *P* = 0.0094, F_group_ = 0.9115, *P* = 0.3511; for rearing time, F_time_ = 6.733, *P* = 0.0104, F_group_ = 0.09130, *P* = 0.7657) **(A)**, grid performance test **(B)**, rotarod test (F_time_ = 68.68, *P* < 0.001, F_group_ = 1.286, *P* = 0.2629) **(C)**, myodynamia test (F_time_ = 18.46, *P* < 0.001, F_group_ = 0.01431, *P* = 0.9060) **(D)**, pole test (for time to start, F_time_ = 38.07, *P* =< 0.001, F_group_ = 0.7477, *P* = 0.3975; for time of the tophalf, F_time_ = 114.6, *P* < 0.001, F_group_ = 0.9545, *P* = 0.3339; for time of bottomhalf, F_time_ = 50.14, *P* < 0.001, F_group_ = 0.05571, *P* = 0.8158) **(E)** of male and female N103 mice at different ages. Mice were grouped by gender. Two-way ANOVA and Bonferroni’s multiple comparisons were used for analysis. Data are mean ± s.e.m.; *n* = 11 male N103 mice of 3 months, *n* = 9 male N103 mice of 9 months, *n* = 7 male N103 mice of 16 months, *n* = 11 female N103 mice of 3 months, *n* = 6 female N103 mice of 9 months, and *n* = 6 female N103 mice of 16 months. **(F)** Fecal analysis showing the excremental water content of N103 mice. Mann–Whitney *U* test was applied.

## Abbreviations

PD: Parkinson’s disease
AEP: asparagine endopeptidase
N103 mice: transgenic mouse line expressing α -synuclein 1-103
LBs: Lewy bodies
LNs: Lewy neurites
SNCA: α -synuclein coding gene
MPTP: 1-methyl-4-phenyl-1,2,3,6-tetrahydropyridine
6-OHDA: 6-hydroxydopamine
UTR: untranslated region
qPCR: quantitative polymerase chain reaction
α -synuclein p-S 129: α -synuclein phosphorylated at Ser129
TH: tyrosine hydroxylase
TEM: transmission electron microscopy
SDAs: synaptic density areas
HPLC: high-performance liquid chromatography
DOPAC: 3,4-dihydroxyphenylacelic acid
HVA: homovanillic acid
DEGs: differentially expressed genes.

## References

[B1] Ahn E. H. Kang S. S. Liu X. Chen G. Zhang Z. Chandrasekharan B. (2020). Initiation of Parkinson’s disease from gut to brain by δ-secretase. *Cell Res.* 30 70–87. 10.1038/s41422-019-0241-9 31649329 PMC6951265

[B2] Anderson J. P. Walker D. E. Goldstein J. M. de Laat R. Banducci K. Caccavello R. J. (2006). Phosphorylation of Ser-129 Is the Dominant Pathological Modification of α-Synuclein in Familial and Sporadic Lewy Body Disease. *J. Biol. Chem.* 281 29739–29752. 10.1074/jbc.M600933200 16847063

[B3] Armstrong M. J. Okun M. S. (2020). Diagnosis and Treatment of Parkinson Disease: a Review. *JAMA* 323:548. 10.1001/jama.2019.22360 32044947

[B4] Balestrino R. Schapira A. H. V. (2020). Parkinson disease. *Eur. J. Neurol.* 27 27–42. 10.1111/ene.14108 31631455

[B5] Blesa J. (2018). Advances in Parkinson’s Disease: 200 Years Later. *Front. Neuroanat.* 12:113. 10.3389/fnana.2018.00113 30618654 PMC6306622

[B6] Braak H. Tredici K. D. Rüb U. de Vos R. A. I. Jansen Steur E. N. H. Braak E. (2003). Staging of brain pathology related to sporadic Parkinson’s disease. *Neurobiol. Aging* 24 197–211. 10.1016/S0197-4580(02)00065-9 12498954

[B7] Bressman S. Saunders-Pullman R. (2019). When to Start Levodopa Therapy for Parkinson’s Disease. *N. Engl. J. Med.* 380 389–390. 10.1056/NEJMe1814611 30673551

[B8] Chesselet M.-F. Richter F. Zhu C. Magen I. Watson M. B. Subramaniam S. R. (2012). A Progressive Mouse Model of Parkinson’s Disease: the Thy1-aSyn (“Line 61”) Mice. *Neurotherapeutics* 9 297–314. 10.1007/s13311-012-0104-2 22350713 PMC3337020

[B9] Daher J. Ying M. Banerjee R. McDonald R. S. Hahn M. Yang L. (2009). Conditional transgenic mice expressing C-terminally truncated human α-synuclein (αSyn119) exhibit reduced striatal dopamine without loss of nigrostriatal pathway dopaminergic neurons. *Mol. Neurodegener.* 4:34. 10.1186/1750-1326-4-34 19630976 PMC2722624

[B10] Decressac M. Ulusoy A. Mattsson B. Georgievska B. Romero-Ramos M. Kirik D. (2011). GDNF fails to exert neuroprotection in a rat α-synuclein model of Parkinson’s disease. *Brain* 134 2302–2311. 10.1093/brain/awr149 21712347

[B11] Fleming S. M. (2004). Early and Progressive Sensorimotor Anomalies in Mice Overexpressing Wild-Type Human -Synuclein. *J. Neurosci.* 24 9434–9440. 10.1523/JNEUROSCI.3080-04.2004 15496679 PMC6730110

[B12] Giasson B. I. Duda J. E. Quinn S. M. Zhang B. Trojanowski J. Q. (2002). Neuronal α-Synucleinopathy with Severe Movement Disorder in Mice Expressing A53T Human α-Synuclein. *Neuron* 34 521–533. 10.1016/S0896-6273(02)00682-7 12062037

[B13] Goedert M. (2015). Alzheimer’s and Parkinson’s diseases: the prion concept in relation to assembled Aβ, tau, and α-synuclein. *Science* 349:1255555. 10.1126/science.1255555 26250687

[B14] Jackson-Lewis V. Przedborski S. (2007). Protocol for the MPTP mouse model of Parkinson’s disease. *Nat. Protoc.* 2 141–151. 10.1038/nprot.2006.342 17401348

[B15] Jagmag S. A. Tripathi N. Shukla S. D. Maiti S. Khurana S. (2016). Evaluation of Models of Parkinson’s Disease. *Front. Neurosci.* 9:503. 10.3389/fnins.2015.00503 26834536 PMC4718050

[B16] Kahle P. J. Neumann M. Ozmen L. Schindzielorz A. Okochi M. Leimer U. (2000). Subcellular Localization of Wild-Type and Parkinson’s Disease-Associated Mutant α-Synuclein in Human and Transgenic Mouse Brain. *J. Neurosci.* 20 6365–6373. 10.1523/JNEUROSCI.20-17-06365.2000 10964942 PMC6772969

[B17] Kellie J. F. Higgs R. E. Ryder J. W. Major A. Beach T. G. Adler C. H. (2015). Quantitative Measurement of Intact Alpha-Synuclein Proteoforms from Post-Mortem Control and Parkinson’s Disease Brain Tissue by Intact Protein Mass Spectrometry. *Sci. Rep.* 4:5797. 10.1038/srep05797 25052239 PMC4107347

[B18] Lewis K. A. Su Y. Jou O. Ritchie C. Foong C. Hynan L. S. (2010). Abnormal Neurites Containing C-Terminally Truncated α-Synuclein Are Present in Alzheimer’s Disease without Conventional Lewy Body Pathology. *Am. J. Pathol.* 177 3037–3050. 10.2353/ajpath.2010.100552 21056999 PMC2993276

[B19] Li W. West N. Colla E. Pletnikova O. Troncoso J. C. Marsh L. (2005). Aggregation promoting C-terminal truncation of α-synuclein is a normal cellular process and is enhanced by the familial Parkinson’s disease-linked mutations. *Proc. Natl. Acad. Sci. U. S. A.* 102 2162–2167. 10.1073/pnas.0406976102 15684072 PMC548541

[B20] Lu X. Kim-Han J. Harmon S. Sakiyama-Elbert S. E. O’Malley K. L. (2014). The Parkinsonian mimetic, 6-OHDA, impairs axonal transport in dopaminergic axons. *Mol. Neurodegener.* 9:17. 10.1186/1750-1326-9-17 24885281 PMC4016665

[B21] Luk K. C. Kehm V. Carroll J. Zhang B. O’Brien P. Trojanowski J. Q. (2012). Pathological α-Synuclein Transmission Initiates Parkinson-like Neurodegeneration in Nontransgenic Mice. *Science* 338 949–953. 10.1126/science.1227157 23161999 PMC3552321

[B22] Masuda-Suzukake M. Nonaka T. Hosokawa M. Oikawa T. Arai T. Akiyama H. (2013). Prion-like spreading of pathological α-synuclein in brain. *Brain* 136 1128–1138. 10.1093/brain/awt037 23466394 PMC3613715

[B23] McKinnon C. De Snoo M. L. Gondard E. Neudorfer C. Chau H. Ngana S. G. (2020). Early-onset impairment of the ubiquitin-proteasome system in dopaminergic neurons caused by α-synuclein. *Acta Neuropathol. Commun.* 8:17. 10.1186/s40478-020-0894-0 32059750 PMC7023783

[B24] McNaught K. S. P. Olanow C. W. Halliwell B. Isacson O. Jenner P. (2001). Failure of the ubiquitin–proteasome system in Parkinson’s disease. *Nat. Rev. Neurosci.* 2 589–594. 10.1038/35086067 11484002

[B25] Nguyen M. Wong Y. C. Ysselstein D. Severino A. Krainc D. (2019). Synaptic, Mitochondrial, and Lysosomal Dysfunction in Parkinson’s Disease. *Trends Neurosci.* 42 140–149. 10.1016/j.tins.2018.11.001 30509690 PMC6452863

[B26] Okun M. S. (2012). Deep-Brain Stimulation for Parkinson’s Disease. *N. Engl. J. Med.* 367 1529–1538. 10.1056/NEJMct1208070 23075179

[B27] Okun M. S. (2017). Management of Parkinson Disease in 2017: personalized Approaches for Patient-Specific Needs. *JAMA* 318:791–792. 10.1001/jama.2017.7914 28828469

[B28] Peelaerts W. Bousset L. Van der Perren A. Moskalyuk A. Pulizzi R. Giugliano M. (2015). α-Synuclein strains cause distinct synucleinopathies after local and systemic administration. *Nature* 522 340–344. 10.1038/nature14547 26061766

[B29] Perier C. Bové J. Vila M. Przedborski S. (2003). The rotenone model of Parkinson’s disease. *Trends Neurosci.* 26 345–346. 10.1016/S0166-2236(03)00144-9 12850429

[B30] Prasad K. Beach T. G. Hedreen J. Richfield E. K. (2012). Critical Role of Truncated α-Synuclein and Aggregates in Parkinson’s Disease and Incidental Lewy Body Disease: role of Truncated α-Synuclein. *Brain Pathol.* 22 811–825. 10.1111/j.1750-3639.2012.00597.x 22452578 PMC5505643

[B31] Recasens A. Dehay B. Bové J. Carballo-Carbajal I. Dovero S. Pérez-Villalba A. (2014). Lewy body extracts from Parkinson disease brains trigger α-synuclein pathology and neurodegeneration in mice and monkeys. *Ann. Neurol.* 75 351–362. 10.1002/ana.24066 24243558

[B32] Reeve A. K. Grady J. P. Cosgrave E. M. Bennison E. Chen C. Hepplewhite P. D. (2018). Mitochondrial dysfunction within the synapses of substantia nigra neurons in Parkinson’s disease. *NPJ Parkinsons Dis.* 4:9. 10.1038/s41531-018-0044-6 29872690 PMC5979968

[B33] Sorrentino Z. A. Giasson B. I. (2020). The emerging role of α-synuclein truncation in aggregation and disease. *J. Biol. Chem.* 295 10224–10244. 10.1074/jbc.REV120.011743 32424039 PMC7383394

[B34] Tieu K. (2011). A Guide to Neurotoxic Animal Models of Parkinson’s Disease. *Cold Spring Harb. Perspect. Med.* 1:a009316. 10.1101/cshperspect.a009316 22229125 PMC3234449

[B35] Tofaris G. K. (2006). Pathological Changes in Dopaminergic Nerve Cells of the Substantia Nigra and Olfactory Bulb in Mice Transgenic for Truncated Human α-Synuclein(1-120): implications for Lewy body disorders. *J. Neurosci.* 26 3942–3950. 10.1523/JNEUROSCI.4965-05.2006 16611810 PMC6673887

[B36] van der Putten H. Wiederhold K.-H. Probst A. Barbieri S. Mistl C. Danner S. (2000). Neuropathology in Mice Expressing Human α-Synuclein. *J. Neurosci.* 20 6021–6029. 10.1523/JNEUROSCI.20-16-06021.2000 10934251 PMC6772584

[B37] Wakamatsu M. Ishii A. Iwata S. Sakagami J. Ukai Y. Ono M. (2008a). Selective loss of nigral dopamine neurons induced by overexpression of truncated human α-synuclein in mice. *Neurobiol. Aging* 29 574–585. 10.1016/j.neurobiolaging.2006.11.017 17174013

[B38] Wakamatsu M. Iwata S. Funakoshi T. Yoshimoto M. (2008b). Dopamine receptor agonists reverse behavioral abnormalities of α-synuclein transgenic mouse, a new model of Parkinson’s disease. *J. Neurosci. Res.* 86 640–646. 10.1002/jnr.21513 17896793

[B39] Zhang L. Yu X. Ji M. Liu S. Wu X. Wang Y. (2018). Resveratrol alleviates motor and cognitive deficits and neuropathology in the A53T α-synuclein mouse model of Parkinson’s disease. *Food Funct.* 9 6414–6426. 10.1039/C8FO00964C 30462117

[B40] Zhang Z. Kang S. S. Liu X. Ahn E. H. Zhang Z. He L. (2017a). Asparagine endopeptidase cleaves α-synuclein and mediates pathologic activities in Parkinson’s disease. *Nat. Struct. Mol. Biol.* 24 632–642. 10.1038/nsmb.3433 28671665 PMC5871868

[B41] Zhang Z. Obianyo O. Dall E. Du Y. Fu H. Liu X. (2017b). Inhibition of delta-secretase improves cognitive functions in mouse models of Alzheimer’s disease. *Nat. Commun.* 8:14740. 10.1038/ncomms14740 28345579 PMC5378956

