# Supplementary figures and images for: Transgenic Mice Expressing Human α-Synuclein 1-103 Fragment as a Novel Model of Parkinson’s Disease
Source: Front Aging Neurosci. 2021 Oct 22;13:760781. doi: 10.3389/fnagi.2021.760781 (PMC8569470; doi:10.3389/fnagi.2021.760781)

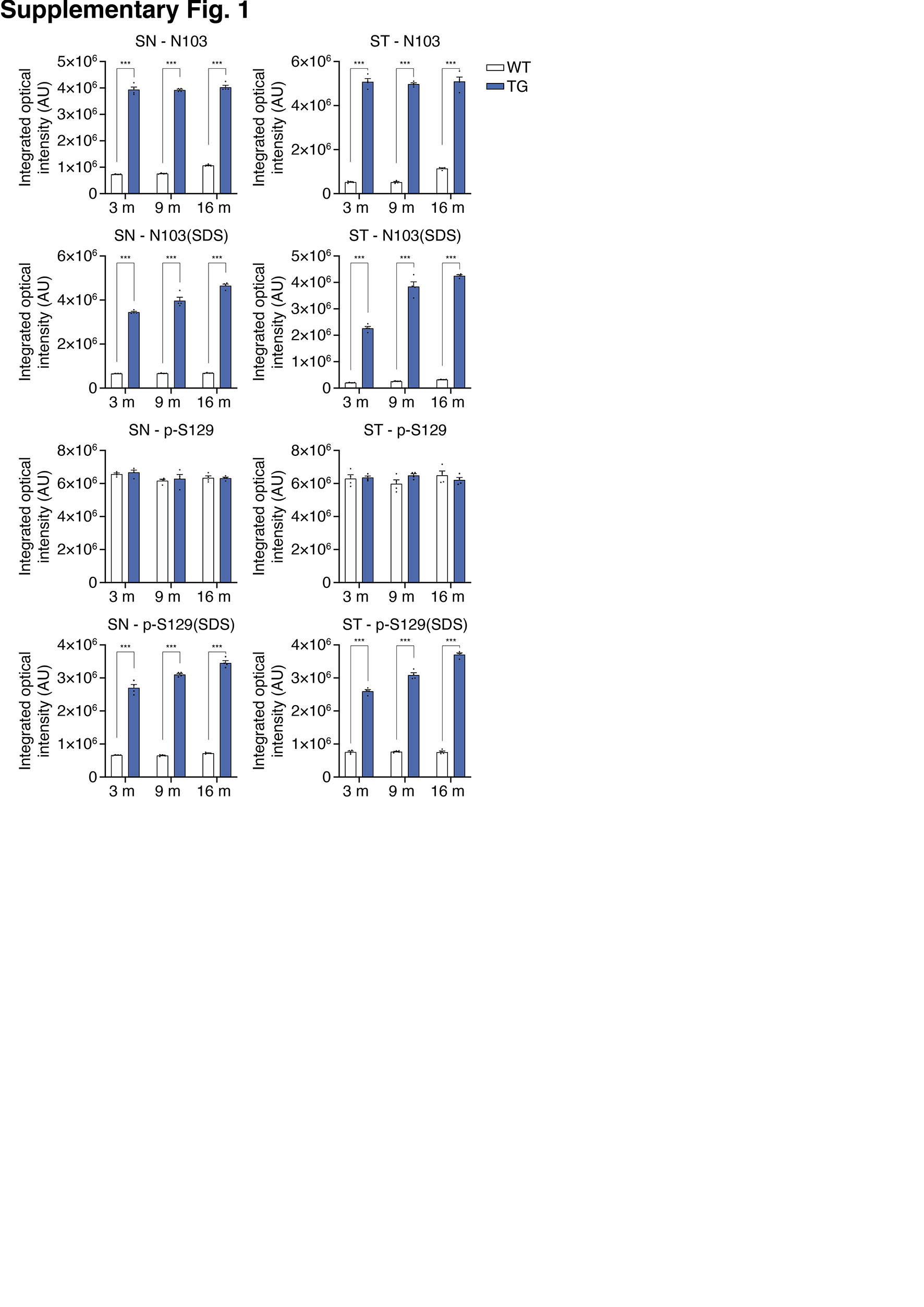

Supplement: Supplementary Figure 1 — Quantification of Western blots showing the soluble and insoluble α-synuclein 1-103 and α-synuclein p-S129 in the substantia nigra and striatum of the mouse brains (expressed as arbitrary units). SN, substantia nigra; ST, striatum. Data are shown as mean ± s.e.m.; n = 4 mice per group; ∗∗∗P < 0.001 by two-way ANOVA and Bonferroni’s multiple comparisons. F and P values are: FSN–N103 Time = 8.389, P = 0.0151, FSN–N103 Group = 11128, P < 0.001; FSN–N103(SDS) Time = 35.69, P < 0.001, FSN–N103(SDS) Group = 3181, P < 0.001; FSN–p–S129 Time = 4.369, P = 0.0284, FSN–p–S129 Group = 0.3896, P = 0.5403; FSN–p–S129(SDS) Time = 31.10, P < 0.001, FSN–p–S129(SDS) Group = 3307, P < 0.001; FST–N103 Time = 7.176, P = 0.0051, FST–N103 Group = 2528, P < 0.001; FST–N103(SDS) Time = 94.40, P < 0.001, FST–N103(SDS) Group = 2365, P < 0.001; FST–p–S129 Time = 0.2137, P = 0.8096, FST–p–S129 Group = 0.3859, P = 0.5423; and FST–p–S129(SDS) Time = 81.04, P < 0.001, FST–p–S129(SDS) Group = 4429, P < 0.001. [file Image_1.JPEG]

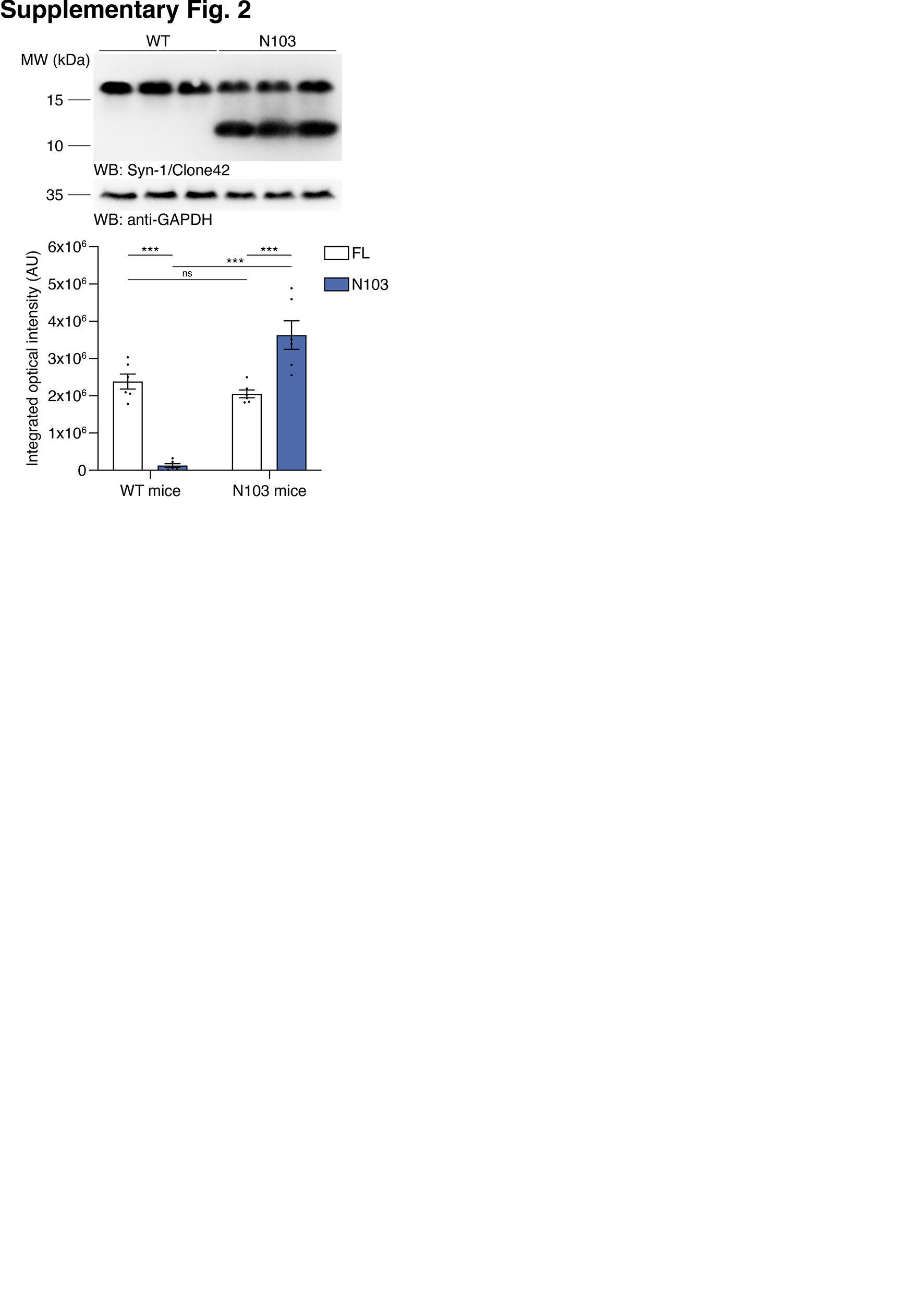

Supplement: Supplementary Figure 2 — Western blots showing the expression of the endogenous mouse α-synuclein and imported α-synuclein 1-103 in WT mice and N103 mice at the age of 9 months. Bar graph, quantification of the Western blot results (expressed as arbitrary units). Data are shown as mean ± s.e.m.; n = 6 mice per group; ∗∗∗P < 0.001 by two-way ANOVA and Bonferroni’s multiple comparisons. F and P values are: FProtein = 2.335, P = 0.1422 and FGroup = 50.56, for all P < 0.001. [file Image_2.JPEG]

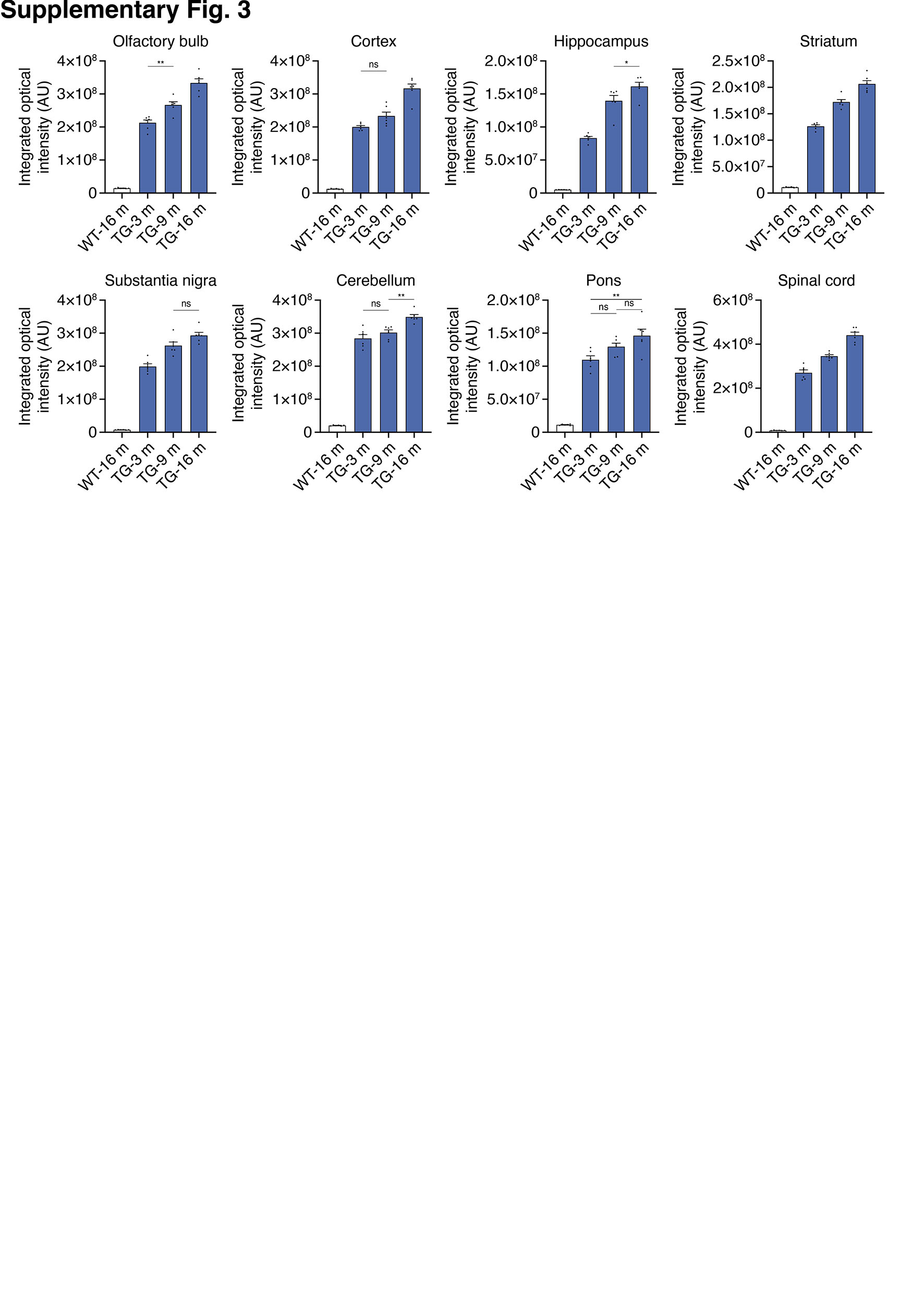

Supplement: Supplementary Figure 3 — Quantification of the IHC results showing α-synuclein 1-103 in the central nervous system of N103 mice (expressed as arbitrary units). Data are shown as mean ± s.e.m.; n = 6 slices per group; ns., non-specific, ∗P < 0.05, and ∗∗P < 0.01; all the unmarked comparisons among these groups mean P < 0.001 by one-way ANOVA and Tukey’s multiple comparisons. F and P values are: FOlfactory bulb = 241.8, FCortex = 191.7, FHippocampus = 176.7, FStriatum = 421.0, FSubstantia nigra = 237.2, FCerebellum = 337.2, FPons = 91.48, and FSpinal cord = 345.9, for all P < 0.001. [file Image_3.JPEG]

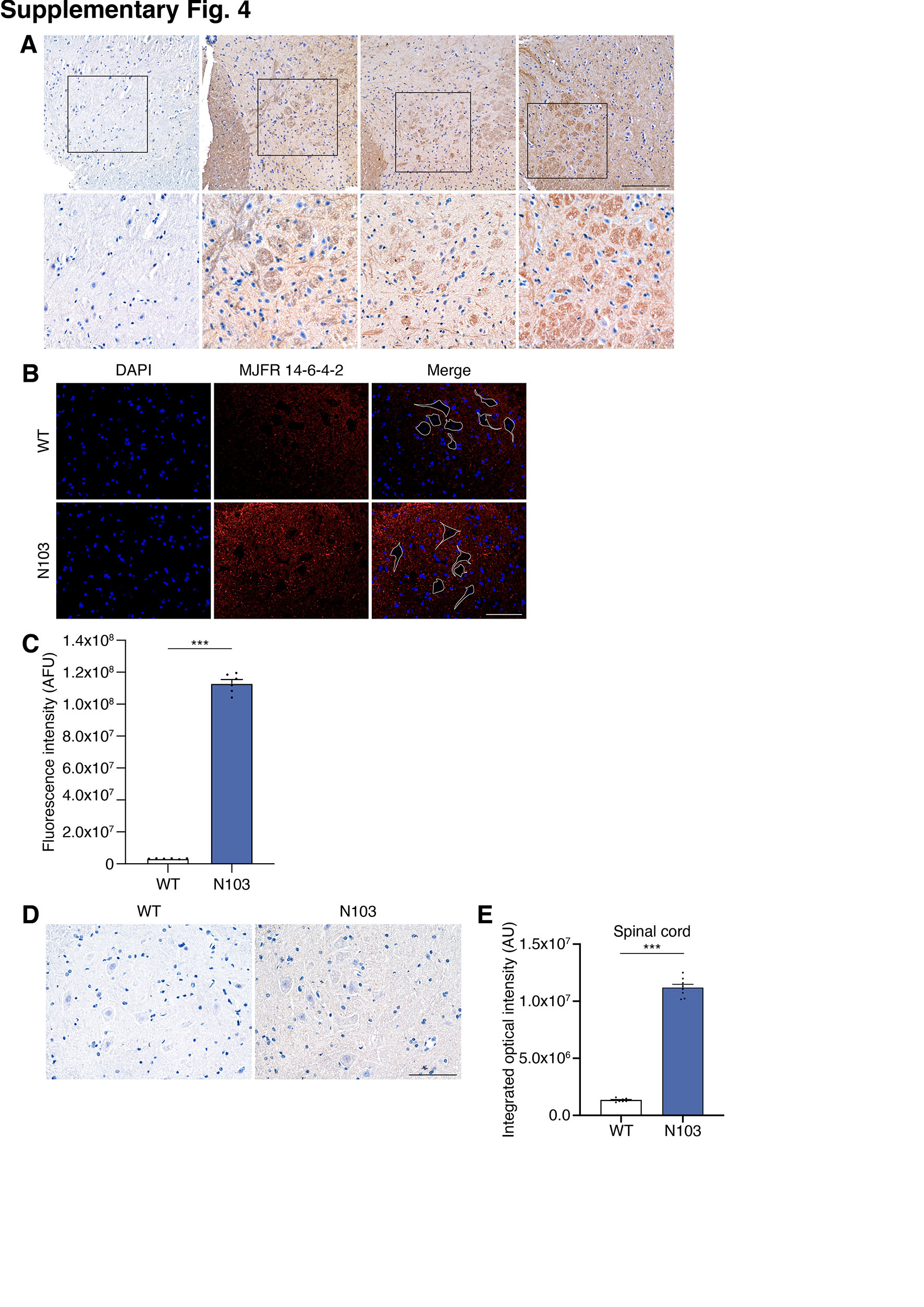

Supplement: Supplementary Figure 4 — Immunohistochemistry and immunofluorescence analysis showing abundant pathological α-synuclein in the spinal cord of N103 mice. (A) α-Synuclein 1-103 fragments in the white matter bundles traveling through the dorsal horn of the spinal cord. Scale bar, 160 μm. Images are zoomed below. (B) α-Synuclein filaments labeled by MJFR 14-6-4-2 antibody in the spinal cord anterior horn of the aged N103 mice. Rare was detected in the age-matched WT mice. Motor neurons were outlined. Scale bar, 80 μm. (C) Quantification of the integrated fluorescence intensity (expressed as arbitrary fluorescence units) of (B). Data are shown as mean ± s.e.m.; n = 6 slices per group; ∗∗∗P < 0.001 by unpaired student t-test (t = 44.26, P < 0.001). (D) Proteinase K digestion showing fine granular hydrolysis-resistant α-synuclein p-S129 in motor neurons in the spinal anterior horn of the aged N103 mice. (E) Quantification analysis of the integrated fluorescence intensity (expressed as arbitrary units) of (D). Data are shown as mean ± s.e.m.; n = 8 slices per group; ∗∗∗P < 0.001 by unpaired student t-test (t = 33.46, P < 0.001). [file Image_4.JPEG]

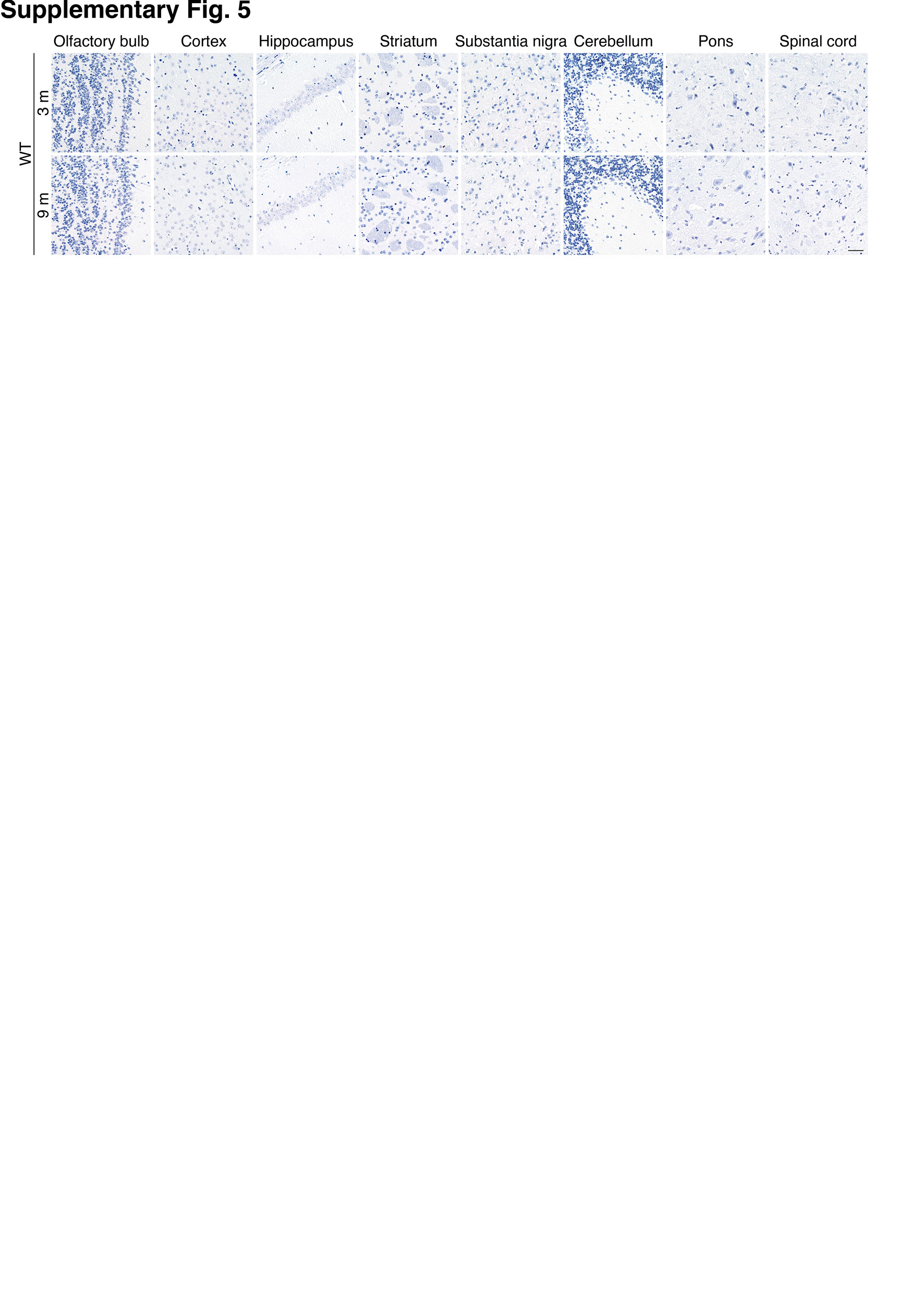

Supplement: Supplementary Figure 5 — Expression of α-synuclein 1-103 fragment in different regions of the central nervous system in the WT mice at the age of 3 and 9 months. Brain sections of WT mice were stained with the anti-N103 antibody. Scale bar, 40 μm. [file Image_5.JPEG]

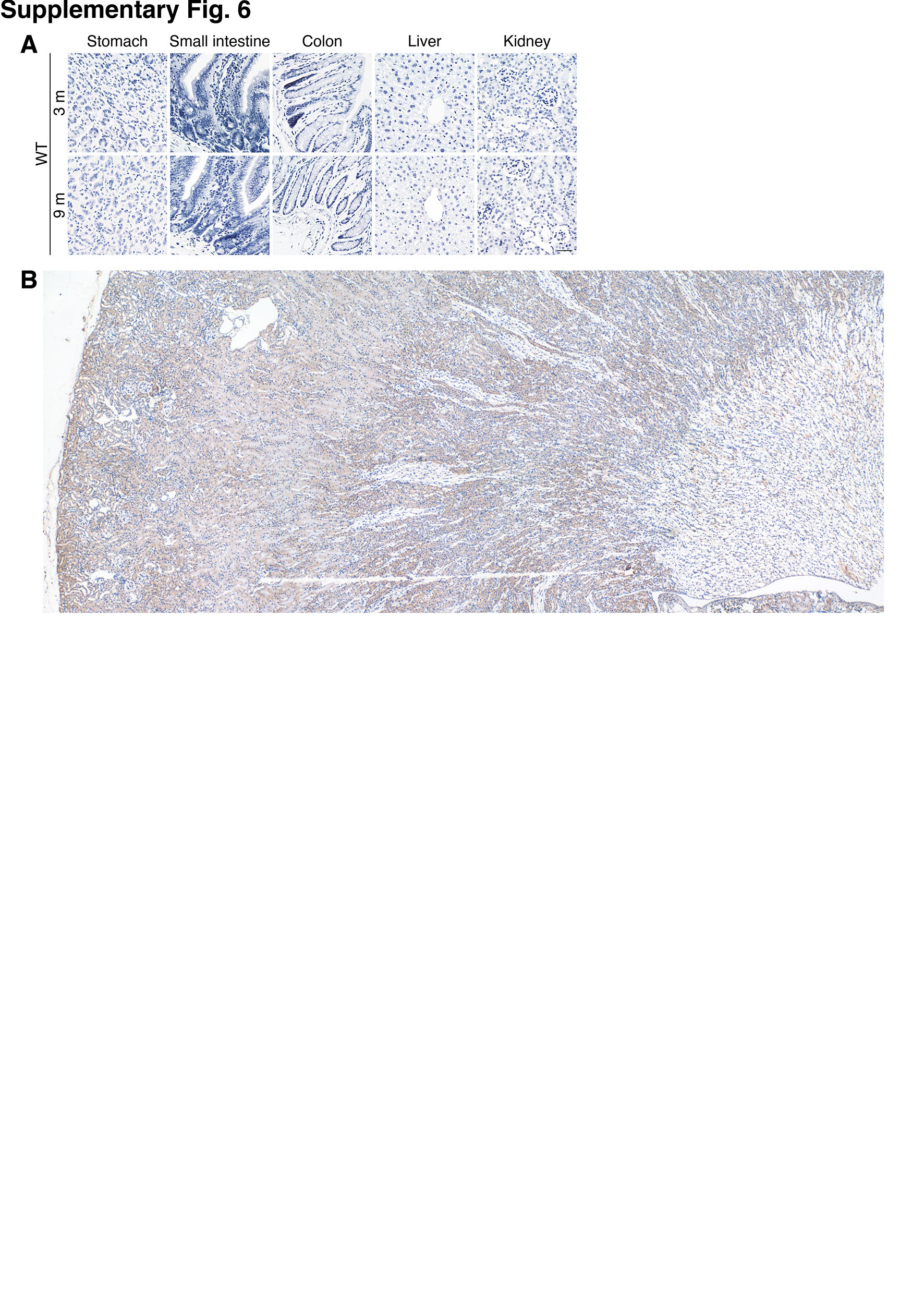

Supplement: Supplementary Figure 6 — The expression of α-synuclein 1-103 in the non-neural tissues of WT mice and N103 mice. (A) α-Synuclein 1-103 in the non-neural tissues of WT mice at different ages. Scale bar, 40 μm. (B) Panorama showing the distribution pattern of α-synuclein 1-103 in the kidney of 9-month-old N103 mice. [file Image_6.JPEG]

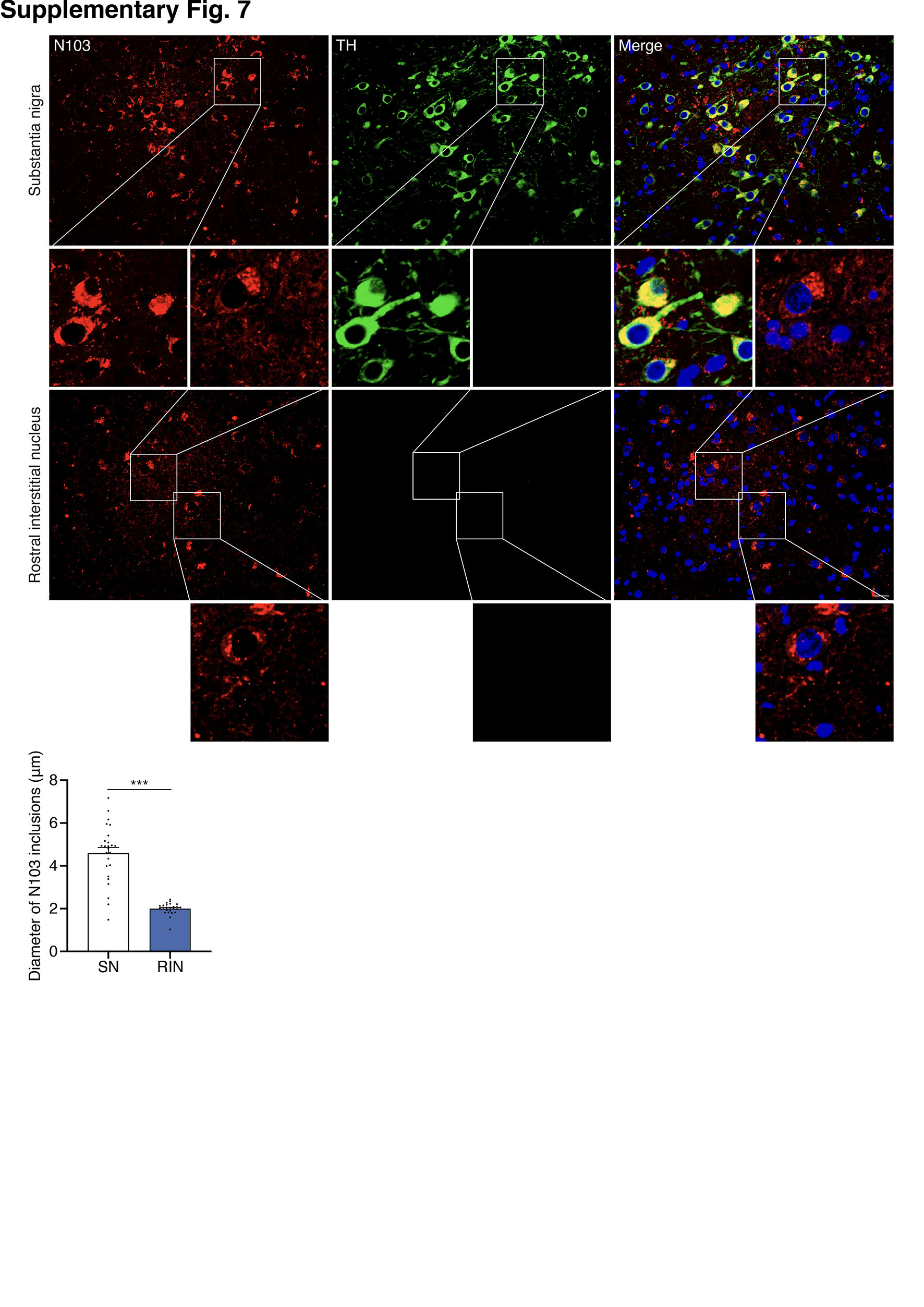

Supplement: Supplementary Figure 7 — α-Synuclein 1-103 is more prone to form compact chunks in the TH-positive cells of the substantia nigra rather than small round pieces in the TH-negative cells. Regions of interest are zoomed. Scale bar, 20 μm. Quantification showing diameter of N103 inclusions in the substantia nigra (SN) and a region near SN, rostral interstitial nucleus (RIN). Data are shown as mean ± s.e.m.; n = 25 slices for SN and n = 24 slices for RIN; ∗∗∗P < 0.001 by unpaired student t-test (4.589 μm vs. 2.000 μm, t = 9.384, P < 0.001). [file Image_7.JPEG]

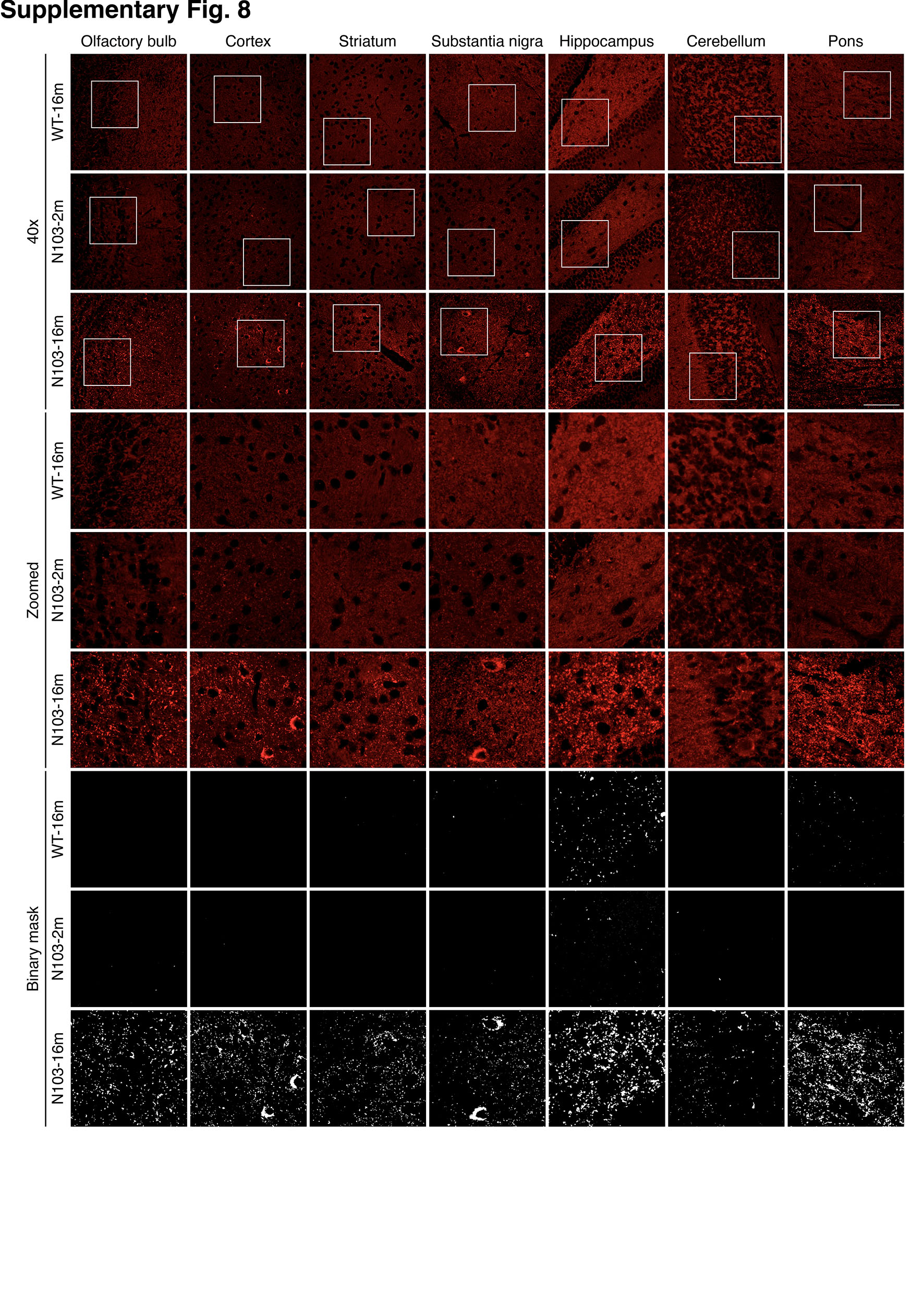

Supplement: Supplementary Figure 8 — Abundant α-synuclein aggregates are detected in the brains of the aged N103 mice. Immunofluorescence using MJFR 14-6-4-2 antibody showing the α-synuclein filaments in different areas of the central nervous system in N103 mice at the age of 2 and 16 months, and 16-month-old WT mice. Scale bar, 80 μm. Regions of interest are zoomed in the middle. The positive staining area is masked by binarization with a proper brightness threshold for baseline correction. The outputs are illustrated below. [file Image_8.JPEG]

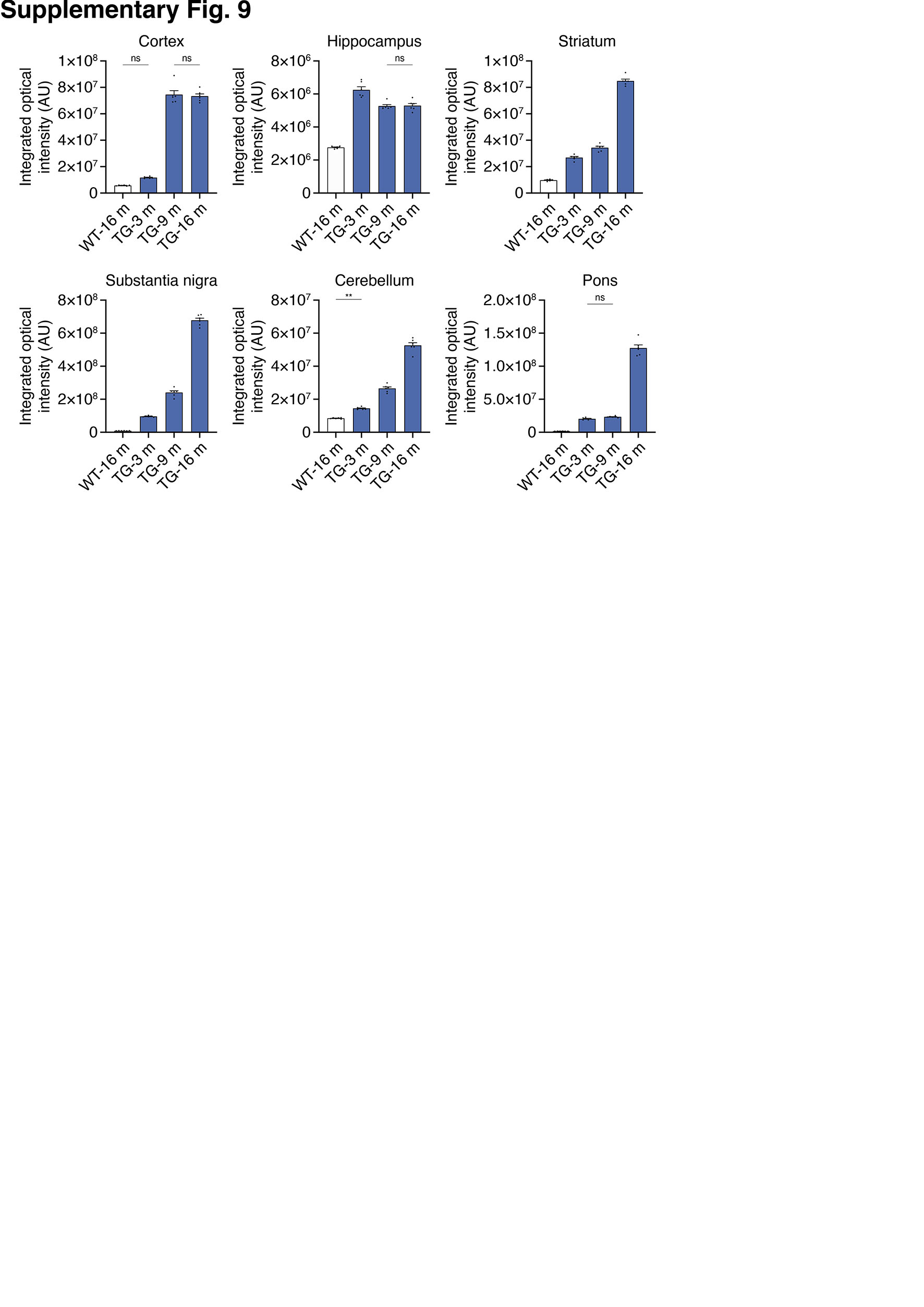

Supplement: Supplementary Figure 9 — Quantification analysis of the accumulated α-synuclein p-S129 in the central nervous system of N103 mice (expressed as arbitrary units). Data are shown as mean ± s.e.m.; n = 6 slices per group; ns., non-specific, ∗∗P < 0.01; all the unmarked comparisons among these groups mean P < 0.001 by one-way ANOVA and Tukey’s multiple comparisons. F and P values are: FCortex = 468.3, FHippocampus = 148.0, FStriatum = 1085, FSubstantia nigra = 1266, FCerebellum = 418.6, and FPons = 583.9, for all P < 0.001. [file Image_9.JPEG]

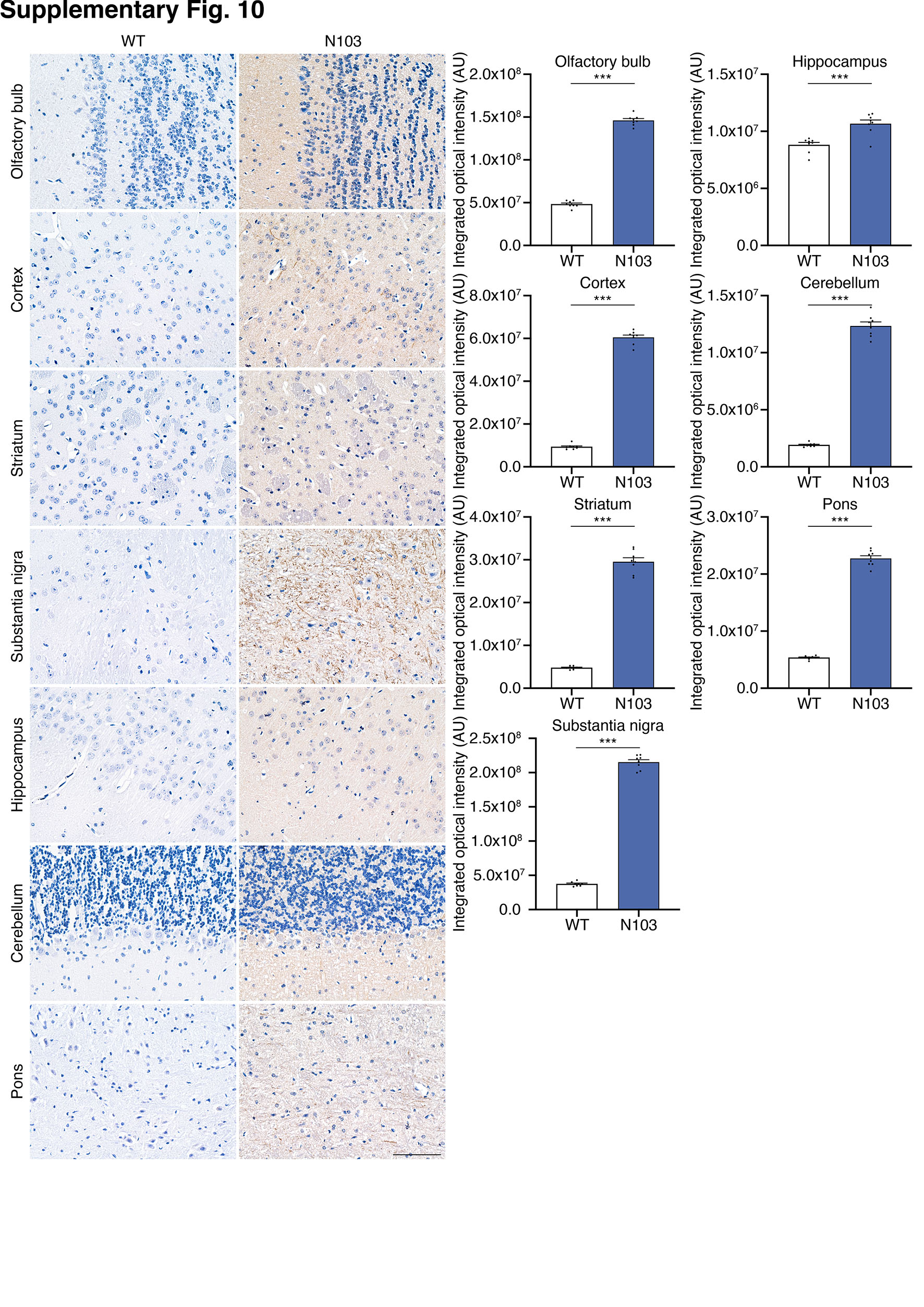

Supplement: Supplementary Figure 10 — Proteinase K digestion showing hydrolysis-resistant α-synuclein p-S129 in the brains of 16-month-old WT mice and N103 mice. Digestion-resistant α-synuclein p-S129 is mainly detected in the cortex, substantia nigra, and pons. Scale bar, 80 μm. Quantification showing the integrated optical intensity (expressed as arbitrary units). Data are shown as mean ± s.e.m.; n = 8 slices per group; ∗∗∗P < 0.001 by unpaired student t-test (tOlfactory bulb = 38.15, tCortex = 43.31, tHippocampus = 4.641, tStriatum = 26.76, tSubstantia nigra = 47.93, tCerebellum = 29.96, and tPons = 34.70, for all P < 0.001). [file Image_10.JPEG]

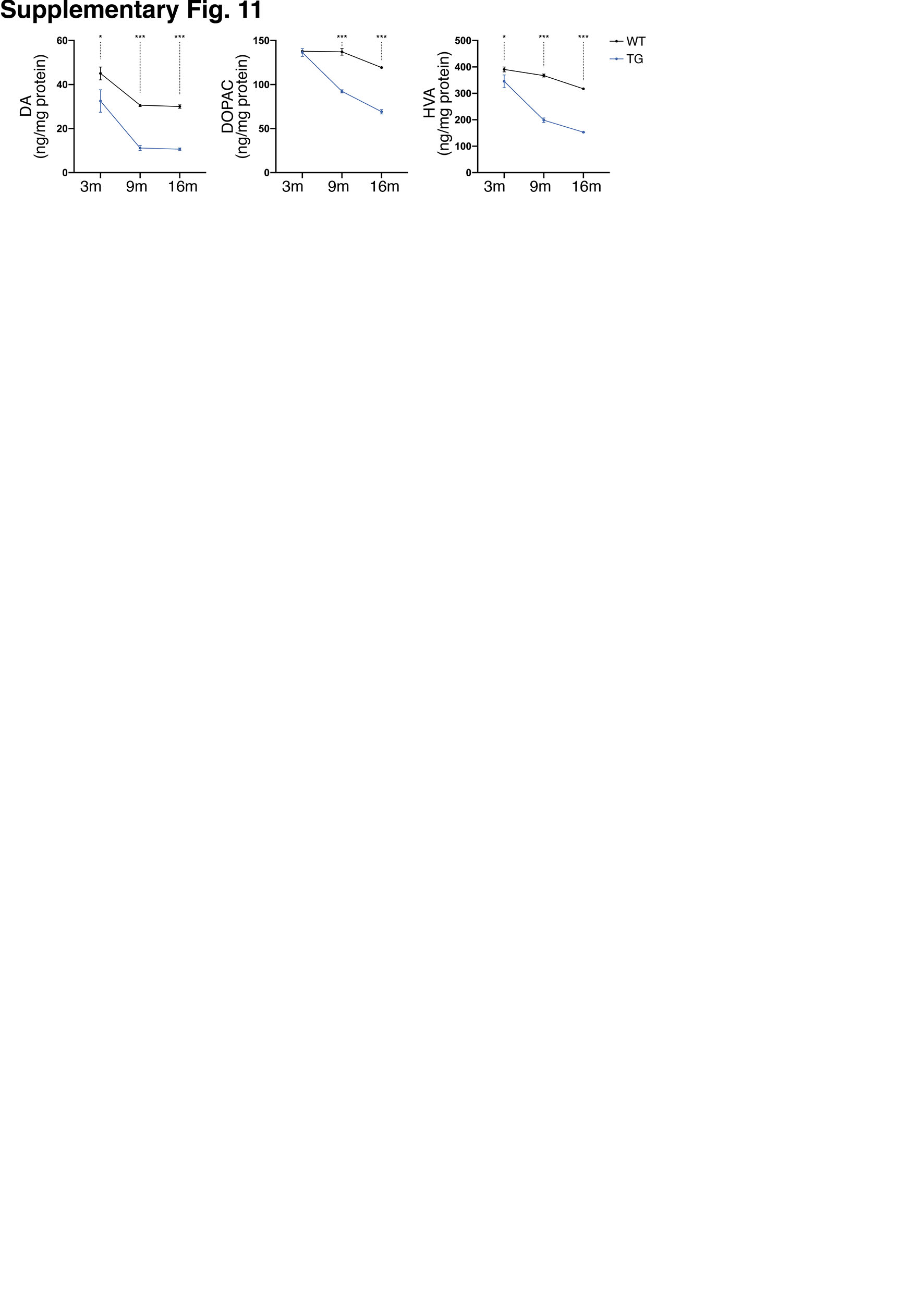

Supplement: Supplementary Figure 11 — HPLC analysis of the striatal neurotransmitters (DA, DOPAC, and HVA) in N103 mice and WT littermates. Results were expressed as absolute contents (ng/mg). Data are mean ± s.e.m.; samples from n = 3 mice per group; ∗P < 0.05 and ∗∗∗P < 0.001 by two-way ANOVA and Bonferroni’s multiple comparisons (for DA, Ftime = 35.56, Fgroup = 70.88; for DOPAC, Ftime = 124.6, Fgroup = 210.1; for HVA, Ftime = 70.38, and Fgroup = 185.7; for all P < 0.001). [file Image_11.JPEG]

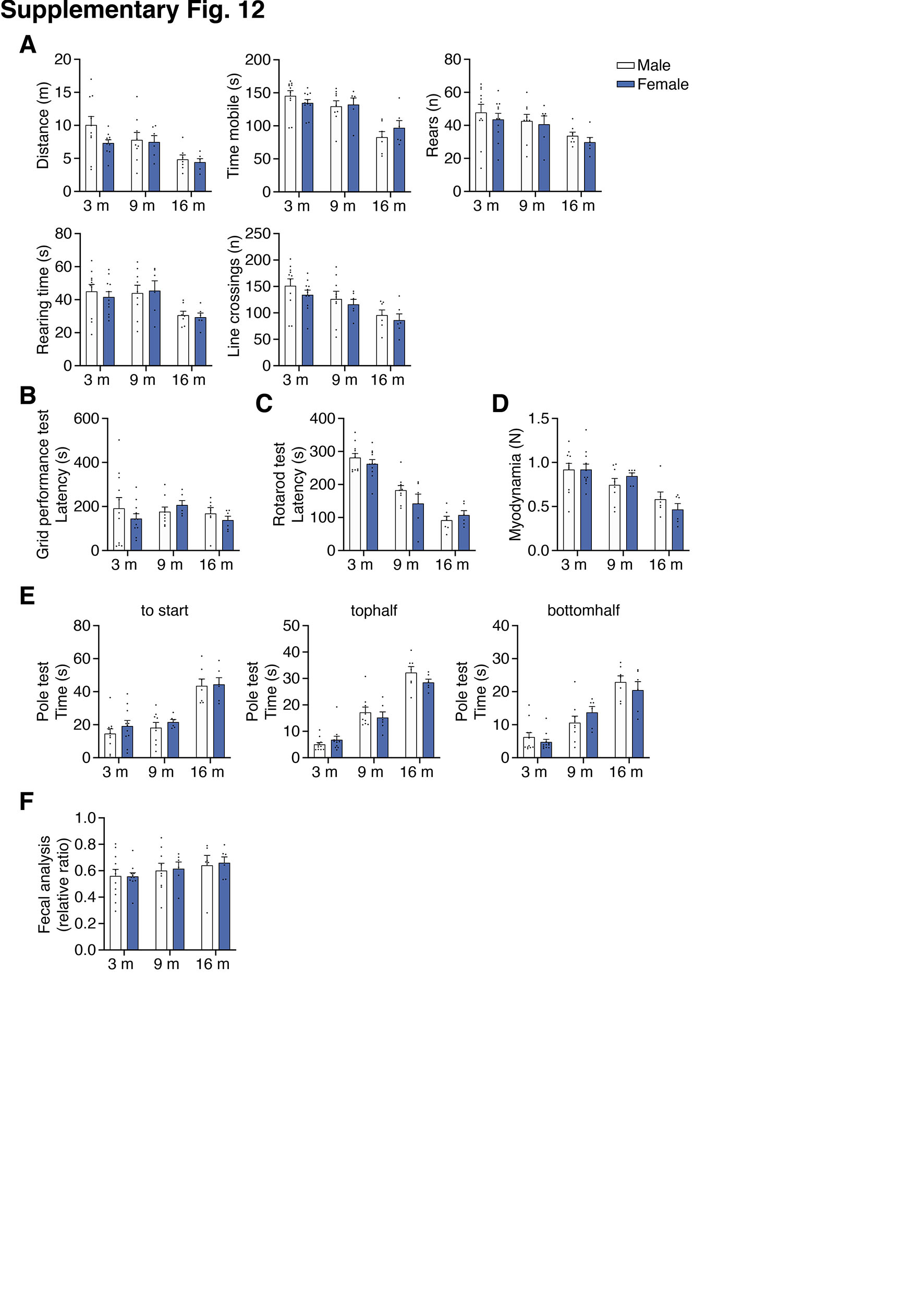

Supplement: Supplementary Figure 12 — Behavioral impairment and constipation of N103 mice do not demonstrate gender differences. Open field test (for distance, Ftime = 8.454, P = 0.0009, Fgroup = 1.922, P = 0.1726; for time mobile, Ftime = 20.27, P < 0.001, Fgroup = 0.06189, P = 0.8061; for line crossings, Ftime = 9.005, P = 0.0006, Fgroup = 1.436, P = 0.2372; for rears, Ftime = 5.877, P = 0.0094, Fgroup = 0.9115, P = 0.3511; for rearing time, Ftime = 6.733, P = 0.0104, Fgroup = 0.09130, P = 0.7657) (A), grid performance test (B), rotarod test (Ftime = 68.68, P < 0.001, Fgroup = 1.286, P = 0.2629) (C), myodynamia test (Ftime = 18.46, P < 0.001, Fgroup = 0.01431, P = 0.9060) (D), pole test (for time to start, Ftime = 38.07, P =< 0.001, Fgroup = 0.7477, P = 0.3975; for time of the tophalf, Ftime = 114.6, P < 0.001, Fgroup = 0.9545, P = 0.3339; for time of bottomhalf, Ftime = 50.14, P < 0.001, Fgroup = 0.05571, P = 0.8158) (E) of male and female N103 mice at different ages. Mice were grouped by gender. Two-way ANOVA and Bonferroni’s multiple comparisons were used for analysis. Data are mean ± s.e.m.; n = 11 male N103 mice of 3 months, n = 9 male N103 mice of 9 months, n = 7 male N103 mice of 16 months, n = 11 female N103 mice of 3 months, n = 6 female N103 mice of 9 months, and n = 6 female N103 mice of 16 months. (F) Fecal analysis showing the excremental water content of N103 mice. Mann–Whitney U test was applied. [file Image_12.JPEG]
